# Synthesis of *S*- and *N*,*S*-Heterocycle–Dipeptide Conjugates for Supramolecular Hydrogel Formation

**DOI:** 10.3390/molecules30040869

**Published:** 2025-02-14

**Authors:** Ana-Morgana G. P. Silva, Maria F. Martins, Carlos B. P. Oliveira, José A. Martins, Paula M. T. Ferreira, Maria-João R. P. Queiroz

**Affiliations:** Centro de Química, Universidade do Minho, Campus de Gualtar, 4710-057 Braga, Portugal; anamorganagpsilva@gmail.com (A.-M.G.P.S.); mariamartinst19@gmail.com (M.F.M.); carlos.oliveira.6b@gmail.com (C.B.P.O.); jmartins@quimica.uminho.pt (J.A.M.); pmf@quimica.uminho.pt (P.M.T.F.)

**Keywords:** *S* and *N*,*S*-heterocycles, dipeptides, *N*-heterocycle–dipeptide conjugates, hydrogels

## Abstract

Small peptides with aromatic nuclei at the *N*-terminus have been shown to form bioactive, biocompatible, and biodegradable supramolecular peptide hydrogels. Novel heterocycle–dipeptide conjugates with potential biological activity or application as drug carriers were synthesized by using *S-*(benzo[*b*]thiophene) and *N*,*S-*(thieno [2,3-*b*]pyridine and thieno[2,3-*b*]quinoline) heterocycles as *N*-protective groups for dipeptides l-Phe-l-Phe and l-Phe-l-Leu. The synthesis involved coupling heterocyclic carboxylic acids with trifluoroacetate salts of ethyl l-phenylalanyl-l-phenylalaninate and ethyl l-phenylalanyl- l-leucinate using HBTU and Et_3_N, producing the corresponding six *N*-heterocycle–dipeptide ester conjugates, which were then hydrolyzed to the carboxylic acids. These conjugates were subjected to gelation tests in water starting from 0.4 wt% concentration of the conjugates, using a pH-lowering method with GdL. Among them, only the conjugate of benzo[*b*]thiophene with l-Phe-l-Phe-OH formed a hydrogel, with a gelation critical concentration of 0.15 wt% (GdL 0.6%) and a final pH of 6.8, which is important for biological applications. The hydrogel was characterized by STEM, revealing nanofibers with an average thickness of 17 nm that assemble into a 3D network capable of trapping water. Further rheological analysis demonstrated its viscoelastic behavior (G′ = 3.03 × 10^3^ Pa; G″ = 3.28 × 10^2^ Pa), comparable to the extracellular matrix of certain human tissues, crucial for biomedical applications.

## 1. Introduction

Peptide-based hydrogels typically have three-dimensional (3D) fibrous networks cross-linked through physical or chemical bonds and are characterized by high-water content, good biocompatibility, tunable mechanical stability, and tissue-like elasticity. The properties of peptide hydrogelators closely mimic those of the extracellular matrix (ECM), and as such, they have found many biomedical applications like drug delivery [1], tissue engineering [2], disease diagnosis [3], wound healing, and bioimaging [4], among others [5]. Peptide hydrogelators offer several advantages, including ease of synthesis, low toxicity, and the ability to fine-tune their mechanical properties by modifying the physical–chemical characteristics of the amino acid side chains and backbone incorporation, allowing specific applications. In the area of drug delivery, self-assembly can be employed for incorporating (by co-assembly) a therapeutic molecule within the hydrogel network. These hydrogels are formed from peptide amphiphiles, which are short peptide sequences modified with lipophilic aliphatic or aromatic groups [6,7].

Small peptides with *N*-terminal aromatic or heteroaromatic capping groups, such as fluorenylmethoxy carbonyl (Fmoc), naphthalene, indole, or benzoimidazole derivatives [8], facilitate self-assembly in aqueous environments. This results in supramolecular hydrogels that are cross-linked through non-covalent interactions like hydrogen bonding, van der Waals forces, and π-π stacking. To obtain effective low-molecular-weight hydrogelators (LMWHs), it is crucial to achieve a balance between hydrophilic and hydrophobic components, which ensures optimal self-assembly properties, allowing the hydrogelators to form stable networks. These *N*-(hetero)aromatic capped peptide conjugate hydrogels combine moderate mechanical strength from polymeric building blocks with a reversible gel–sol transition that can be triggered by various stimuli, such as pH changes, redox agents, enzymes, and bioactive molecules. The supramolecular cross-linking of the physical hydrogels also enhances processability, making these hydrogels promising for applications in controlled drug delivery systems and as scaffolds for tissue and organ repair and regeneration [9,10]. Their preparation offers advantages over nanoparticle-incorporated hydrogels, as some formulation methods are complex, and sometimes, the nanoparticles interfere with gel formation [11].

**Figure 1:**
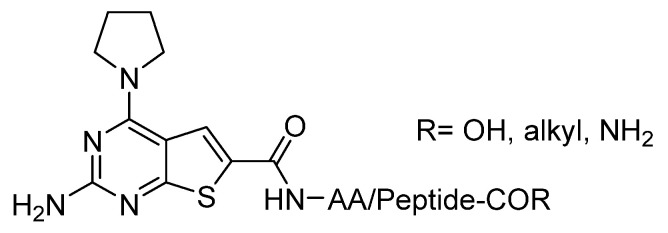
Peptidomimetics obtained by coupling 2-amino-4-pyrrolidinothieno [2,3-*d*]pyrimidine-6-carboxylic acid to the *N*-terminus of different amino acids or peptides [14].

In recent years, the field of peptidomimetics—compounds designed to mimic the key structural features (pharmacophore) of natural peptides or proteins—has gained significant attention for the development of non-natural peptide analogs with enhanced biological activity. One effective strategy involves the linkage of heterocyclic systems with peptides, which imposes conformational constraints, resulting in heterocycle–peptide conjugates with improved in vivo efficacy compared to natural peptides. Additionally, heterocyclic systems can be introduced to impart their unique biological and physicochemical properties to peptidomimetics, further enhancing their therapeutic potential [12,13]. In 2005, Varvounis, Cordopatis et al. prepared 2-amino-4-pyrrolidinothieno [2,3-*d*]pyrimidine-6-carboxylic acid as an *N*-terminal surrogate for the development of *N*-heterocyclic amino acid and peptide conjugates, aimed at producing potential biologically active peptidomimetics (Figure 1) [14].

**Figure 2:**
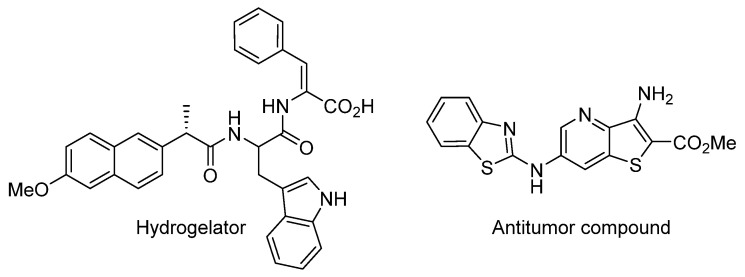
Structures of carrier dehydrodipeptide Npx-l-Trp-*Z*-ΔPhe-OH and antitumor compound methyl 3-amino-6-(benzo[*d*]thiazol-2-ylamino)thieno [3,2-*b*]pyridine-2-carboxylate [15].

In 2015, some of us prepared dipeptides containing tryptophan *N*-capped with naproxen (Npx), a non-steroidal anti-inflammatory drug (NSAID), and *C*-terminal dehydroamino acids, which provide efficient, protease-resistant hydrogelators. Characterization by transmission electron microscopy (TEM) revealed that the hydrogels consisted of networks of micro/nanosized fibers produced by peptide self-assembly. Rheologic experiments demonstrated that the dipeptide containing dehydrophenylalanine (ΔPhe) produced the most elastic gels, achieving gelation at lower critical concentrations than those containing dehydroalanine (ΔAla) or dehydroaminobutyric acid (ΔAbu) [15]. A diheteroarylamine derivative of thieno [3,2-*b*]pyridine, also synthesized by our team, demonstrated promising antitumor activity against various human tumor cell lines [16]. This was non-covalently incorporated into a hydrogel containing a *C*-terminal ∆Phe (hydrogelator) (Figure 2). Fluorescence and Förster resonance energy transfer (FRET) measurements revealed that the diheteroarylamine was located within a hydrophobic environment, closely associated with the peptide fibers, highlighting the potential of the hydrogel as an effective drug nanocarrier [15].

**Figure 3:**
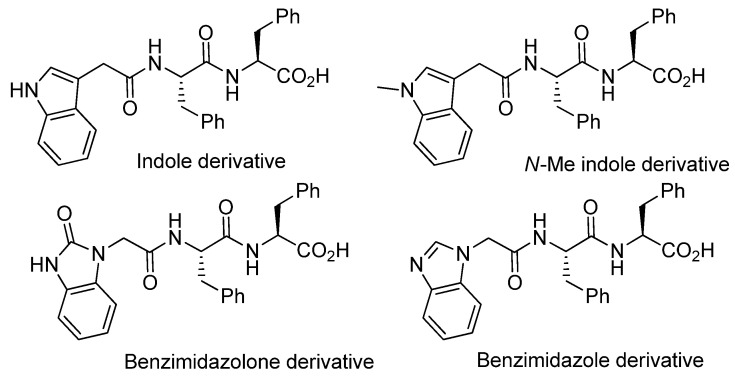
Structures of four diphenylalanine-based peptides *N*-capped with different heterocycle derivatives [8].

In 2016, Martin, Thordarson et al. prepared four diphenylalanine-based peptides bearing *N*-heterocyclic capping groups (indole, *N*-methylindole, benzimidazolone and benzimidazole derivatives) with different degrees of nitrogen substitution and hydrogen bond potential (Figure 3). All four peptides formed hydrogels via a pH switch mechanism using glucono-δ-lactone (GdL), where the final pH of the gel is approximately 4–5. The minimum gelation concentrations were 0.3, 0.6, 0.02, and 0.1 wt%, respectively. The authors conclude that the structure of the *N*-capping group affects both the self-assembly of the dipeptide and the mechanical properties of the hydrogel [8].

The use of other *N*-capping heterocycles in peptide-based hydrogels is therefore an interesting area to explore for the rational design of functional hydrogels for future biomedical applications.

In this work, benzo[*b*]thiophene, thieno[2,3-*b*]pyridine, and thieno[2,3-*b*]quinoline nuclei were chosen as *N*-capping groups for the dipeptides synthesized, aiming to create new *N*-heterocycle–dipeptide conjugates. These conjugates were then subjected to gelation and rheological analyses to assess their potential for forming hydrogels for biological applications.

## 2. Results and Discussion

### 2.1. Synthesis of Dipeptides N-Capped with Benzo[b]Thiophene, Thieno[2,3-b]Pyridine, or Thieno[2,3-b]Quinoline

**Scheme 1:**
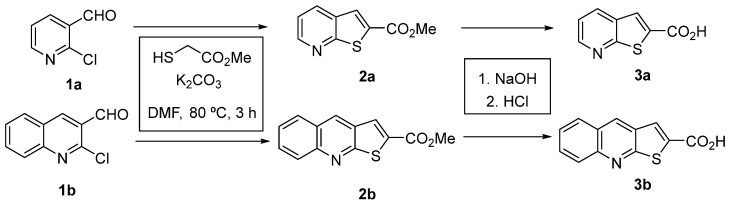
Synthesis of the heterocyclic esters **2a**,**b** and the corresponding carboxylic acids **3a**,**b**.

The reaction of 2-chloropyridine-3-carbaldehyde (**1a**) or 2-chloroquinoline-3-carbaldehyde (**1b**) with methyl thioglycolate produced the heterocyclic methyl esters **2a** and **2b** using K_2_CO_3_ as a base in DMF heating at 80 °C for 3 h [17,18]. These compounds were subsequently hydrolyzed to the respective carboxylic acids **3a** and **3b**, using NaOH 2 M in MeOH overnight at room temperature (r.t.). The resulting mixtures were then acidified with concentrated HCl, yielding the carboxylic acids in quantitative yields (Scheme 1) [17,19].

**Scheme 2:**
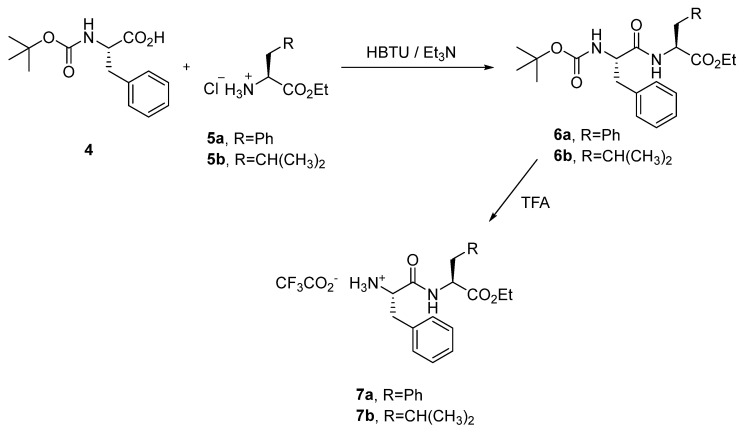
Synthesis of ethyl esters of *N*-deprotected dipeptides **7a** and **7b**.

*N*-(*tert*-butoxycarbonyl)-l-phenylalanine (Boc-Phe-OH) (**4**) was coupled with the ethyl esters of l-phenylalanine **5a** and l-leucine (Leu) **5b** using HBTU as the coupling agent [20]. The resulting *N*,*C*-diprotected dipeptides **6a** and **6b** were subsequently treated with trifluoroacetic acid (TFA) to remove the Boc protecting group, yielding the ethyl esters of the *N*-deprotected dipeptides l-phenylalanyl-l-phenylalanine (H-Phe-Phe-OEt) **7a** and l-phenylalanyl-l-leucine (H-Phe-Leu-OEt) **7b** in quantitative yields (Scheme 2).

**Scheme 3:**
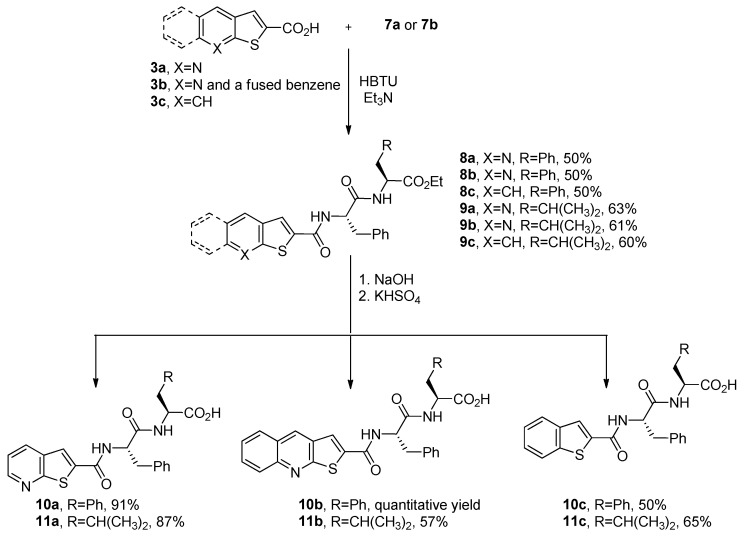
Synthesis of dipeptides *N*-capped with thiophene based heterocycles.

The trifluoroacetate salts dipeptides **7a** or **7b** were reacted with thieno[2,3-*b*]pyridine-2-carboxylic acid **3a**, thieno[2,3-*b*]quinolone-2-carboxylic acid **3b**, or the commercially available benzo[*b*]thiophene-2-carboxylic acid **3c** in the presence of HBTU and Et_3_N to afford the corresponding ethyl esters of *N*-capped dipeptides **8a**–**c** and **9a**–**c** in good yields (50–63%). Subsequent basic hydrolysis of these produced the corresponding carboxylic acids **10a**–**c** and **11a**–**c** in good to quantitative yields (Scheme 3).

### 2.2. Hydrogelation

LMWHs self-organize through multiple non-covalent interactions which enable the monomeric blocks to associate into fibrillar structures. These fibrils interlace and interact with each other, forming the 3D network of the hydrogel.

**Figure 4:**
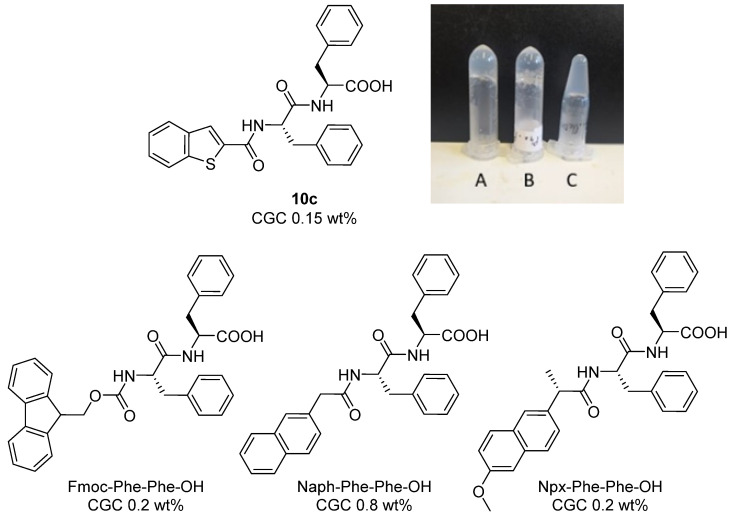
Gelation of compound **10c**, **A** (0.15 wt%), **B** (0.3 wt%), and **C** (0.4 wt%), along with the structures and the literature-reported CGC of l-phenylalanyl-l-phenylalanine *N*-protected with fluorenylmethoxycarbonyl (Fmoc) [24], 2-naphthylacetyl (Naph) [26], and naproxen (Npx) [27] groups.

The hydrogelation capabilities of conjugates **10a**–**c** and **11a**–**c** were tested using pH and temperature triggers. In all the conditions tested, only compound **10c** afforded self-standing hydrogels using a pH trigger (Figure 4). The critical gelation concentration (CGC) was 0.15 wt%, and the final pH was 6.8. The hydrogel was obtained by dissolving compound **9c** in water at pH 10 and lowering the pH by adding GdL which is hydrolyzed to gluconic acid. The dipeptide phenylalanylalphenylalanine (H-Phe-Phe-OH) forms hydrogels at a CGC of 0.25 wt% using a solvent-switch method with ultrasounds [21] and at 6.0 wt% (pH 7.3) with a pH-triggered approach [22]. *N*-heteroaromatic Phe-Phe-OH derivatives form hydrogels with low CGC values using a pH trigger [23], namely indole (0.3 wt%) and benzimidazole (0.1 wt%) derivatives mentioned in the introduction [8], which compare well with the low CGC of compound **10c**.

The Phe-Phe dipeptides *N*-capped with the well-known fluorenylmethoxycarbonyl (Fmoc) group form hydrogels at a CGC of 0.2 wt% [24,25], while the CGC for the *N*-2-naphthylacetyl (Naph) conjugate is 0.8 wt% [26], using a pH trigger. The CGC for the *N*-naxopren (Nxp) conjugate is 0.2 wt% by the heating/cooling method [27] (Figure 4).

However, our findings suggest that the inclusion of *N*-capping groups containing thienopyridine or thienoquinoline moieties hinders hydrogel formation. These *N*-capping groups may become protonated under certain conditions, potentially interfering with the self-assembly process essential for hydrogelation.

**Figure 5:**
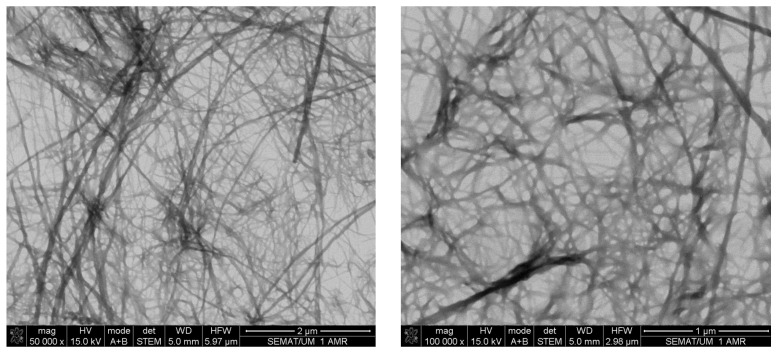
STEM images of the hydrogel formed by compound **10c** (0.15 wt%) with GdL (0.6 wt%).

Scanning transmission electron microscopy (STEM) images of the hydrogel of **10c** showed nanofibers with an average thickness of 17 nm that entangle to form a 3D network that entraps water (Figure 5).

**Figure 6:**
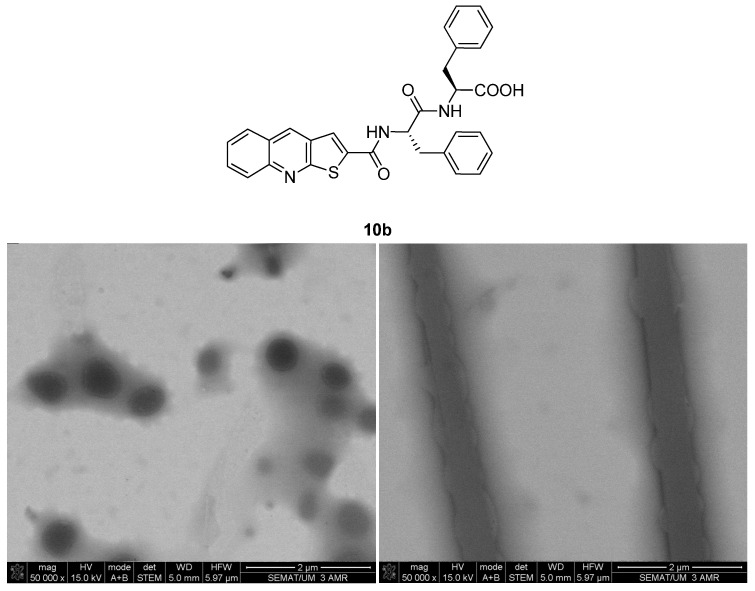
STEM images of the solution of compound **10b** (0.4 wt%) with GdL (0.6 wt%) in water.

Although compounds **10a**, **10b,** and **11a** and **11b** failed to produce hydrogels, it was decided to investigate the possible self-assembly of a dipeptide *N*-protected with a thieno [2,3-*b*]quinoline moiety. Thus, compound **10b** was studied using STEM. The images show that it aggregates to form vesicle-like nanostructures that appear to come together to form fibers. However, the number of fibers is not enough to interlace and form a hydrogel (Figure 6).

### 2.3. Rheology

**Figure 7:**
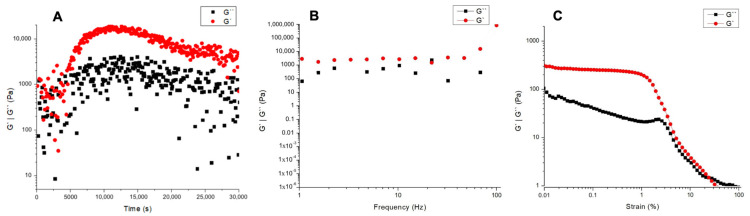
Rheological characterization of the hydrogel formed from compound **10c** at 0.4 wt%. (**A**) Elastic (G′) and viscous (G″) moduli during the kinetics process of hydrogelation. (**B**) Frequency dependence of the shear elastic G′ and G″ moduli (**C**) Strain dependence of the shear elastic G′ and loss G″ moduli.

Rheological studies provide information on the deformation and fluidity of the material under the application of stress and make it possible to evaluate the deformation kinetics of the hydrogel and the moduli G′ (elasticity) and G″ (viscosity) as a function of the force applied. The viscoelastic properties of the hydrogel derived from compound **10c** were investigated using rheological analysis. From the gelation kinetics graphic (Figure 7A), it is possible to conclude that the gel of **10c** requires 6 h to form at a concentration of 0.4 wt%. Once the structural equilibrium established by the G′ and G″ modules was reached, a frequency sweep was performed from 100 Hz to 0.1 Hz, with a strain of 0.01%, to obtain the mechanical spectrum shown in Figure 7B. The G′ value (3.03 × 10^3^ Pa) is approximately 10 times higher than the G″ value (3.28 × 10^2^ Pa). The G′ value is similar to that described for the Nxp-Phe-Phe-OH (G′ 2.1 × 10^3^ Pa) [27]. Next, a strain sweep was performed at a frequency of 1 Hz, increasing the strain from 0.01 to 100%, and the graph shown in Figure 7C was obtained. In a hydrogel, the G′ modulus remains constant, regardless of the applied strain, until the critical strain is reached, beyond which the hydrogel deforms, causing both G′ and G″ values to decrease. This hydrogel has a low critical strain. 

Rheological analysis of the hydrogel formed from compound **10c** revealed viscoelastic behavior (G′ = 3.03 × 10^3^ Pa; G″ = 3.28 × 10^2^ Pa), resembling the extracellular matrix of certain human tissues. This feature is important for its potential use in biological applications.

## 3. Materials and Methods

### 3.1. Synthesis

Melting points (°C) were determined in an SMP3 Stuart apparatus. ^1^H and ^13^C NMR spectra (see Supplementary Materials) were recorded on a Bruker Advance III (Bruker, Bremen, Germany) at 400 and 100.6 MHz, respectively and using TopSpin 2.1 software. DEPT θ = 135° and bi- dimensional homo ^1^H-^1^H (COSY) and heteronuclear correlations ^1^H-^13^C (HSQC and HMBC) were used to attribute some signals. HRMS were obtained at the external service of mass spectrometry of CACTI in the University of Vigo using a mass spectrometer Bruker FTMS SolariX XR for ESI in the [M + H]^+^. The reactions were monitored by thin-layer chromatography (TLC).

#### 3.1.1. Synthesis of Heterocyclic Precursors

##### Methyl Thieno[2,3-*b*]Pyridine-2-Carboxylate (**2a**)

To 2-chloropyridine-3-carbaldehyde (**1a**) (0.650 g, 4.57 mmol) in dry DMF (5.2 mL), anhydrous K_2_CO_3_ (1.900 g, 13.70 mmol) and methyl thioglycolate (0.450 mL, 5.03 mmol) were added. The reaction was left stirring at 80 °C. The reaction was completed after 3 h, and monitoring was carried out by TLC. After cooling, the reaction mixture was poured into ice and stirred, and a precipitate came out. It was filtered under vacuum, washed with water, and dried in the oven at 50 °C to produce compound **2a** as a white solid (0.530 g, 60%). ^1^H NMR (400 MHz, CDCl_3_) δ = 3.97 (3 H, s, OCH_3_), 7.37 (1 H, dd, *J* = 8.0 e 4.4 Hz, 5-H), 8.01 (1 H, s, 3-H), 8.16 (1 H, dd, *J* = 8.0, and 1.6 Hz, 4-H), 8.69 (1 H, dd, *J* = 4.4, and 1.6 Hz, 6-H) ppm. The ^1^H NMR spectrum for compound **1a** was already described in DMSO-*d*_6_ [17].

##### Methyl Thieno[2,3-*b*]Quinoline-2-Carboxylate (**2b**)

To 2-chloroquinoline-3-carbaldehyde (**1b**) (0.350 g, 1.83 mmol) in dry DMF (15 mL), anhydrous K_2_CO_3_ (0.759 mg, 5.49 mmol) and methyl thioglycolate (0.291 g, 0.245 mL, 2.75 mmol) were added. The reaction was left stirring at 80 °C. The reaction was completed after 3 h and monitored by TLC. After cooling, the reaction mixture was poured into ice, stirred, and a precipitate came out. It was filtered under vacuum, washed with water, and dried in the oven at 50 °C to produce compound **2b** as a beige solid (0.288 g, 65%). ^1^H NMR (400 MHz, CDCl_3_) δ = 4.01 (3 H, s, OCH_3_), 7.60–7.66 (1 H, m, ArH), 7.83–7.88 (1 H, m, ArH), 8.03 (1 H, broad d, *J* = 8.4 Hz, ArH), 8.14 (1 H, s, ArH), 8.25 (1 H, broad d, *J* = 8.4 Hz, ArH), 8.75 (1 H, s, ArH) ppm. The ^1^H NMR spectrum for compound **1b** is identical to that reported in the literature [18].

##### Thieno[2,3-*b*]Pyridine-2-Carboxylic Acid (**3a**)

Compound **2a** was dissolved in MeOH (20 mL), and NaOH 2 M (2.0 equiv., 5.48 mmol, 2.75 mL) was added. The reaction was left stirring at room temperature overnight. After partial removal of the solvents, the mixture was acidified using HCl_conc_. until pH = 1, and the precipitate came out. The solid was filtered under vacuum, washed with H_2_O, and dried in the oven at 50 °C, producing compound **3a** in quantitative yield as a white solid. ^1^H NMR (400 MHz, DMSO-*d*_6_): δ = 7.52 (1 H, dd, *J* = 8.2, and 4.4 Hz, 5-H), 8.11 (1 H, s, 3-H), 8.40 (1 H, dd, *J* = 8.2, and 1.6 Hz, 4-H), 8.69 (1 H, dd, *J* = 4.4, and 1.6 Hz, 6-H) ppm. The ^1^H NMR spectrum of compound **3a** is identical to the one presented in the literature [17].

##### Thieno[2,3-*b*]Quinoline-2-Carboxylic Acid (**3b**)

Compound **2b** was dissolved in MeOH (18 mL)/H_2_O (5.40 mL), and NaOH 2 M (2.15 equiv., 2.79 mmol, 1.40 mL) was added. The reaction was left stirring at room temperature overnight. After partial removal of the solvents, the mixture was acidified using HCl_conc_. until pH = 1, and the precipitate came out. The solid was filtered under vacuum, washed with H_2_O, and dried in the oven at 50 °C, producing compound **3b** in quantitative yield as a white solid. ^1^H NMR (400 MHz, DMSO-*d*_6_): δ = 7.64–7.68 (1 H, m, Ar-H), 7.85–7.90 (1 H, m, Ar-H), 8.09–8.11 (1 H, m, Ar-H), 8.18–8.20 (1 H, m, Ar-H), 8.24 (1 H, s, HetAr-H), 9.05 (1 H, s, HetAr-H) ppm. The ^1^H NMR spectrum of compound **3b** was already described in CDCl_3_ [19].

#### 3.1.2. General Procedure for the Synthesis of Ethyl *N*-(*Tert*-Butoxicarbonyl)-l-phenylalanyl-l-pheylalaninate (**6a**) and Ethyl *N*-(*Tert*-Butoxicarbonyl)-l-phenylalanyl-l-leucinate (**6b**)

The mixture of *N*-(*tert*-butoxicarbonyl)-l-phenylalanine (**4**) (1 mmol) in CH_3_CN (10 mL) was left stirring for some time. HBTU (1.2 equiv.), was added, followed by the hydrochlorides of H-Phe-OEt **5a** or H-Leu-OEt **5b** (1 equiv.). To this mixture, Et_3_N (4 equiv.) was added, and it was left stiring at r.t. overnight. The CH_3_CN was removed under reduced pressure, and AcOEt (20 mL) was added. The washes of the organic phase were carried out using KHSO_4_ 1 M (3 × 15 mL), NaHCO_3_ 1 M (3 × 15 mL), and brine (3 × 15 mL). The organic phase was separated, dried (MgSO_4_), and filtered. Removal of the solvent afforded the *N*-Boc dipeptide ethyl esters **6a** or **6b** in 80% and 72%, respectively.

#### 3.1.3. General Procedure for the Synthesis of Trifluoroacetate Salt of Dipeptides **7a** or **7b**

To *N*-Boc dipeptide ethyl esters **6a** or **6b** (0.500 mmol), TFA (1.50 mL) was added, and the mixture was left stirring at r.t. for 2 h. After that, TFA was removed under reduced pressure, adding diethyl ether several times. The salts of trifluoroacetate of ethyl l-phenylalanyl-l-phenylalaninate (**7a**) and trifluoroacetate of ethyl l-phenylalanyl-l-leucinate (**7b**) were obtained in quantitative yields as white solids.

#### 3.1.4. General Procedure for the Synthesis of Ethyl *N*-Heterocycle Dipeptide Esters **8a**–**c** and **9a**–**c**

To the heterocyclic carboxylic acid **3a**, **3b**, or **3c** (1.0 equiv.), CH_3_CN was added, and the mixture was left stirring for 10 min. The trifluoroacetate salt **7a** or **7b** (1.0 equiv.) was dissolved in CH_3_CN (2–5 mL), and the solution was added to the initial mixture. Using an ice bath with NaCl, at 0 °C, the coupling agent HBTU (1.2 equiv.) was then added and Et_3_N until pH = 7. The mixture was left stirring at r.t. overnight. The CH_3_CN was removed under reduced pressure, and AcOEt (20 mL) was added to the residue. The organic phase was washed with KHSO_4_ 1 M (3 × 15 mL), NaHCO_3_ 1 M (3 × 15 mL), and brine (3 × 15 mL) and separated. It was dried (MgSO_4_) and filtered, and the removal of AcOEt produced a light yellow solid, that after washes with some diethyl ether, afforded the ethyl *N*-heterocycle dipeptide esters **8a**–**c** and **9a**–**c** as white solids.

##### Ethyl *N*-(Thieno[2,3-*b*]Pyridine-2-Carbonyl)-l-phenylalanyl-l-phenylalaninate (**8a**)

From compound **3a** (0.0890 g, 0.500 mmol) in CH_3_CN (15 mL) and trifluoroacetate salt **7a**, following the general procedure, compound **8a** was obtained (0.126 g, 50%), m.p. 135–137 °C. ^1^H NMR (400 MHz, DMSO-*d_6_*) δ = 1.08 (3 H, t, *J* = 7.2 Hz, OCH_2_CH_3_), 3.00–3.07 (4 H, m, 2 × β-CH_2_), 4.02 (2 H, q, *J* = 7.2 Hz, OCH_2_CH_3_), 4.47–4.50 (1 H, m, α-CH), 4.75–4.76 (1 H, m, α-CH), 7.13–7.28 (8 H, m, ArH), 7.33–7.36 (2 H, m, ArH), 7.48 (1 H, dd, *J* = 8.0, and 4.8 Hz, 5-H), 8.15 (1 H, s, 3-H), 8.37 (1 H, dd, *J* = 8.0, and 1.6 Hz, 4-H), 8.63 (1 H, dd, *J* = 4.8, and 1.6 Hz, 6-H), 8.67 (1 H, d, *J* = 7.6 Hz, NH), 9.05 (1 H, d, *J* = 8.8 Hz, NH) ppm. ^13^C NMR (100.6 MHz, DMSO-*d*_6_) δ = 13.9 (CH_3_), 36.6 (β-CH_2_), 37.0 (β-CH_2_), 53.9 (α-CH), 54.6 (α-CH), 60.5 (CH_2_), 120.5 (5-CH), 123.3 (3-CH), 126.3 (CH), 126.5 (CH), 128.1 (CH), 128.2 (CH), 129.1 (CH), 132.6 (C), 133.4 (4-CH), 137.1 (C), 138.1 (C), 139.0 (C), 148.6 (6-CH), 161.0 (C), 162.0 (C), 171.2 (C), 171.3 (C) ppm. HRMS (ESI) [M + H]^+^, *m*/*z* calculated for C_28_H_28_N_3_O_4_S: 502.1795; found: 502.1802.

##### Ethyl *N*-(Thieno[2,3-*b*]Quinoline-2-Carbonyl)-l-phenylalanyl-l-phenylalaninate (**8b**)

From compound **3b** (0.115 g, 0.500 mmol) in CH_3_CN (20 mL) and trifluoroacetate salt **6a**, and following the general procedure, compound **8b** was obtained (0.137 g, 50%), m.p. 196–198 °C. ^1^H NMR (400 MHz, DMSO-*d*_6_) δ = 1.10 (3 H, t, *J* = 6.8 Hz, OCH_2_CH_3_), 2.97–3.25 (4 H, m, 2 × β-CH_2_), 4.03 (2 H, q, *J* = 6.8 Hz, OCH_2_CH_3_), 4.49–4.51 (1 H, m, α-CH), 4.75–4.90 (1 H, m, α-CH), 7.15–7.39 (10 H, m, ArH), 7.62–7.66 (1 H, m, ArH), 7.81–7.86 (1 H, m, ArH), 8.05–8.06 (1 H, br d, ArH), 8.15–8.16 (1 H, br d, ArH), 8.27 (1 H, s, ArH), 8.67 (1 H, d, *J* = 7.2 Hz, NH), 9.04 (1 H, s, ArH), 9.10 (1 H, d, *J* = 8.4 Hz, NH) ppm. ^13^C NMR (100.6 MHz, DMSO-*d_6_*) δ = 13.9 (CH_3_), 36.5 (β-CH_2_), 37.1 (β-CH_2_), 54.0 (α-CH), 54.5 (α- CH), 60.5 (CH_2_), 123.2 (CH), 125.5 (C), 125.8 (CH), 126.3 (CH), 126.5 (CH), 127.8 (CH), 128.1 (CH), 128.2 (CH), 129.0 (CH), 129.1 (CH), 129.12 (CH), 130.5 (CH), 131.7 (C), 133.3 (CH), 137.0 (C), 138.0 (C), 140.0 (C), 147.0 (C), 161.0 (C), 162.0 (C), 171.2 (C) ppm. HRMS (ESI) [M + H]^+^, *m*/*z* calculated for C_32_H_30_N_3_O_4_S: 552.1952; found: 552.1953.

##### Ethyl *N*-(Benzo[*b*]Thiophene-2-Carbonyl)-l-phenylalanyl-l-phenylalaninate (**8c**)

From compound **3c** (0.890 g, 0.500 mmol) in CH_3_CN (15 mL) and trifluoroacetate salt **7a**, following the general procedure, compound **8c** was obtained (0.125 g, 50%), m.p. 120–122 °C. ^1^H NMR (400 MHz, DMSO-*d_6_*) δ = 1.09 (3 H, t, *J* = 7.2 Hz, OCH_2_CH_3_), 2.94–3.07 (4 H, m, 2 × β-CH_2_), 4.03 (2 H, q, *J* = 7.2 Hz, OCH_2_CH_3_), 4.45–4.51 (1 H, m, α-CH), 4.71–4.77 (1 H, m, α-CH), 7.14–7.26 (8 H, m, ArH), 7.34–7.36 (2 H, m, ArH), 7.42–7.44 (2 H, m, ArH), 7.93–8.00 (2 H, m, ArH), 8.15 (1 H, s, 3-H), 8.60 (1 H, d, *J* = 7.6 Hz, NH), 8.87 (1 H, d, *J* = 8.8 Hz, NH) ppm. ^13^C NMR (100.6 MHz, DMSO-*d_6_*) δ = 14.0 (CH_3_), 36.6 (β-CH_2_), 37.0 (β-CH_2_), 54.0 (α-CH), 54.5 (α-CH), 60.5 (CH_2_), 122.7 (CH), 124.9 (CH), 125.1 (3-CH), 125.2 (CH), 126.3 (CH), 126.5 (CH), 128.1 (CH), 128.2 (CH), 129.1 (CH), 137.0 (C), 138.1 (C), 139.1 (C), 139.4 (C), 140.2 (C), 161.3 (C), 171.2 (C), 171.4 (C) ppm. HRMS (ESI) [M + H]^+^, *m*/*z* calculated for C_29_H_29_N_2_O_4_S: 501.1843; found: 501.1849.

##### Ethyl *N*-(Thieno [2,3-*b*]Pyridine-2-Carbonyl)-l-phenylalanyl-l-leucinate (**9a**)

From compound **3a** (0.128 g, 0.713 mmol) in CH_3_CN (8 mL) and trifluoroacetate salt **7b**, and following the general procedure, compound **9a** was obtained (0.210 g, 63%), m.p. 210–212 °C. ^1^H NMR (400 MHz, DMSO-*d_6_*) δ = 0.86 (3 H, d, *J* = 6.4 Hz, CH_3_), 0.91 (3 H, d, *J* = 6.4 Hz, CH_3_) 1.17 (3 H, t, *J* = 7.2 Hz, OCH_2_CH_3_), 1.48–1.73 (3 H, m, CH and β-CH_2_ Leu), 2.94–3.00 (1 H, m, β-CH Phe), 3.11–3.16 (1 H, m, β-CH Phe), 4.02–4.14 (2 H, m, OCH_2_CH_3_), 4.26–4.32 (1 H, m, α-CH Leu), 4.73–4.79 (1 H, m, α-CH Phe), 7.13–7.17 (1 H, m, ArH), 7.23–7.27 (2 H, m, ArH), 7.37–7.39 (2 H, m, ArH), 7.47 (1 H, *J* = 8.0, and 4.8 Hz, 5-H), 8.16 (1 H, s, 3-H), 8.38 (1 H, dd, *J* = 8.0, and 1.6 Hz, 4-H), 8.56 (1 H, d, *J* = 7.6 Hz, NH Leu), 8.62 (1 H, dd, *J* = 4.8, and 1.6 Hz, 6-H), 9.03 (1 H, d, *J* = 8.4 Hz, NH Phe) ppm. ^13^C NMR (100.6 MHz, DMSO-*d_6_*) δ = 14.0 (OCH_2_CH_3_), 21.4 (CH_3_), 22.7 (CH_3_), 24.3 (CH Leu), 37.1 (β-CH_2_ Phe), 39.6 (β-CH_2_ Leu), 50.6 (α-CHLeu), 54.6 (α-CHPhe), 60,5 (OCH_2_CH_3_), 120.5 (5-CH), 123.3 (3-CH), 126.3 (CH), 128.1 (2 × CH), 129.1 (2 × CH), 132.6 (C), 133.4 (4-CH), 138.1 (C), 139.0 (C), 148.6 (6-CH), 161.1 (C), 161.2 (C), 171.4 (C), 172.3 (C) ppm. HRMS (ESI) [M + H]^+^, *m*/*z* calculated for C_25_H_30_N_3_O_4_S: 468.1952; found: 468.1958.

##### Ethyl *N*-(Thieno [2,3-*b*]Quinoline-2-Carbonyl)-l-phenylalanyl-l-leucinate (**9b**)

From compound **3b** (0.330 g, 0.785 mmol) in CH_3_CN (10 mL) and trifluoroacetate salt **7b**, and following the general procedure, compound **9b** was obtained (0.249 g, 61%), m.p. 189–191 °C. ^1^H NMR (400 MHz, DMSO-*d_6_*) δ = 0.86 (3 H, d, *J* = 6.4 Hz, CH_3_), 0.92 (3 H, d, *J* = 6.4 Hz, CH_3_), 1.17 (3 H, t, *J* = 6.8 Hz, OCH_2_CH_3_), 1.48–1.73 (3 H, m, CH and β-CH_2_ Leu), 2.97–3.03 (1 H, m, β-CH Phe), 3.14–3.18 (1 H, m, β-CH Phe), 4.03–4.14 (2 H, m, OCH_2_CH_3_), 4.28–4.34 (1 H, m, α-CH Leu), 4.75–4.90 (1 H, m, α-CH Phe), 7.14–7.18 (1 H, m, ArH), 7.25- 7.29 (2 H, m, ArH), 7.40–7.42 (2 H, m, ArH), 7.61–7.65 (1 H, m, ArH), 7.81–7.85 (1 H, m, ArH), 8.06 (1 H, br d, *J* = 7.6 Hz, ArH), 8.16 (1 H, br d, *J* = 7.6 Hz, ArH), 8.29 (1 H, s, ArH), 8.59 (1 H, d, *J* = 7.6 Hz, NH Leu), 9.03 (1 H, s, ArH), 9.13 (1 H, d, *J* = 8.0 Hz, NH Phe) ppm. ^13^C NMR (100.6 MHz, DMSO-*d_6_*) δ = 14.0 (OCH_2_CH_3_), 21,3 (CH_3_), 22.7 (CH_3_), 24.3 (CHLeu), 37.1 (β-CH_2_ Phe), 39.6 (β-CH_2_ Leu), 50.6 (α-CH Leu), 54.6 (α-CH Phe), 60.5 (OCH_2_CH_3_), 123.2 (CH), 125.5 (C), 125.8 (CH), 126.3 (CH), 127.8 (CH), 128.1 (2 × CH), 128.9 (CH), 129.1 (2 × CH), 130.5 (CH), 131.7 (C), 133.3 (CH), 138.1 (C), 140.0 (C), 146.9 (C), 161.1 (C), 162.0 (C), 171.4 (C), 172.3 (C) ppm. HRMS (ESI) [M + H]^+^, *m*/*z* calculated for C_29_H_32_N_3_O_4_S: 518.2108; found: 518.2109.

##### Ethyl *N*-(Benzo[*b*]Thiophene-2-Carbonyl)-l-phenylalanyl-l-leucinate (**9c**)

From compound **3c** (0.120 g, 0.644 mmol) in CH_3_CN (7 mL) and trifluoroacetate salt **7b**, and following the general procedure, compound **9c** was obtained (0.242 g, 60%), m.p. 212–214 °C. ^1^H NMR (400 MHz, DMSO-*d_6_*) δ = 0.85 (3 H, d, *J* = 6.4 Hz, CH_3_), 0.93 (3 H, d, *J* = 6.4 Hz, CH_3_), 1.17 (3 H, t, *J* = 7.2 Hz, OCH_2_CH_3_), 1.50–1.73 (3 H, m, CH, and β-CH_2_ Leu), 2.94–3.00 (1 H, m, β-CH Phe), 3.11–3.15 (1 H, m, β-CH Phe), 4.02–4.14 (2 H, m, OCH_2_CH_3_), 4.26–4.32 (1 H, m, α-CH Leu), 4.72–4.78 (1 H, m, α-CH Phe), 7.13–7.17 (1 H, m, ArH), 7.23–7.27 (2 H, m, ArH), 7.37–7.39 (2 H, m, ArH), 7.41–7.46 (2 H, m, ArH), 7.92–7.98 (2 H, m, ArH), 8.17 (1 H, s, 3-H), 8.53 (1 H, d, *J* = 7.6 Hz, NH Leu), 8.90 (1 H, d, *J* = 8.8 Hz, NH Phe) ppm. ^13^C NMR (100.6 MHz, DMSO-*d_6_*) δ = 14.0 (OCH_2_CH_3_), 21.4 (CH_3_), 22.7 (CH_3_), 24.3 (CH Leu), 37.1 (β-CH_2_ Phe), 39.6 (β-CH_2_ Leu), 50.6 (α-CH Leu), 54.6 (α-CHPhe), 60.5 (OCH_2_CH_3_), 122.8 (CH), 124.9 (CH), 125.2 (CH), 125.3 (3-CH), 126.2 (CH), 126.3 (CH), 128.1 (2 × CH), 129.1 (2 × CH), 138.2 (C), 139.1 (C), 139.5 (C), 140.2 (C), 161.4 (C), 171.5 (C), 172.3 (C) ppm. HRMS (ESI) [M + H]^+^, *m*/*z* calculated for C_26_H_31_N_2_O_4_S: 467.1999; found: 467.1998.

#### 3.1.5. General Procedure for the Ethyl Ester Hydrolysis of Conjugates **8a**–**c** and **9a**–**c** to the Corresponding Carboxylic Acids **10a**–**c** and **11a**–**c**

From compounds **8a**–**c** and **9a**–**c** (1.0 equiv.) in EtOH, NaOH (2 M) was added until pH = 10–12, and the mixture was stirred at r.t. overnight. The solvent was removed under reduced pressure, H_2_O was added, and then the mixture was acidified to pH = 1 with KHSO_4_ (1 M). A precipitated came out, and it was filtered under vacuum to afford compounds **10a**–**c** and **11a**–**c** as white solids.

##### *N*-(Thieno[2,3-*b*]Pyridine-2-Carbonyl)-l-phenylalanyl-l-phenylalanine (**10a**)

From compound **8a** (0.0860 g, 0.171 mmol) in EtOH (10 mL), following the general procedure, compound **10a** was obtained (0.0740 g, 91%), m.p. 235–237 °C. ^1^H NMR (400 MHz, DMSO-*d_6_*) δ = 2.93–2.99 (2 H, m, β-CH_2_) 3.07–3.12 (2 H, m, β-CH_2_), 4.42–4.48 (1 H, m, α-CH), 4.71–4.77 (1 H, m, α-CH), 7.14–7.25 (8 H, m, ArH), 7.33–7.35 (2 H, m, ArH), 7.47 (1 H, dd, *J* = 8.0, and 4.8 Hz, 5-H), 8.13 (1 H, s, 3-H), 8.36–8.41 (2 H, m, 4-H, and NH), 8.62 (1 H, dd, *J* = 4.8, and 1.6 Hz, 6-H), 8.99 (1 H, d, *J* = 8.8 Hz, NH) ppm. ^13^C NMR (100.6 MHz, DMSO-*d_6_*) δ = 36.6 (β-CH_2_), 37.0 (β-CH_2_), 53.8 (α-CH), 54.7 (α-CH), 120.6 (5-CH), 123.3 (3-CH), 126.3 (CH), 126.4 (CH), 128.1 (CH), 128.2 (CH), 129.15 (CH), 129.2 (CH), 132.6 (C), 133.5 (4-CH), 137.5 (C), 138.1 (C), 138.9 (C), 148.6 (6-CH), 161.0 (C), 162.0 (C), 171.0 (C), 172.7 (C) ppm. HRMS (ESI) [M + H]^+^, *m*/*z* calculated for C_26_H_24_N_3_O_4_S: 474.1482; found: 474.1485.

##### *N*-(Thieno[2,3-*b*]Quinoline-2-Carbonyl)-l-phenylalanyl-l-phenylalanine (**10b**)

From compound **8b** (0.0680 g, 0.123 mmol) in EtOH (10 mL), following the general procedure, compound **10b** was obtained (0.0640 g, quantitative yield), m.p. 206–208 °C. ^1^H NMR (400 MHz, DMSO-*d_6_*) δ = 2.93–3.00 (2 H, m, β-CH_2_), 3.09–3.13 (2 H, m, β-CH_2_), 4.44–4.49 (1 H, m, α-CH), 4.75–4.81 (1 H, m, α-CH), 7.14–7.37 (8 H, m, ArH), 7.36–7.37 (2 H, m, ArH), 7.62–7.66 (1 H, m, ArH), 7.82–7.86 (1 H, m, ArH), 8.07 (1 H, br d, *J* = 8.8 Hz, ArH), 8.17 (1 H, br d, *J* = 8.0 Hz, ArH), 8.26 (1 H, s, ArH), 8.44 (1 H, d, *J* = 7.2 Hz, NH), 9.04 (1 H, s, ArH), 9.10 (1 H, *J* = 8.4 Hz, NH) ppm. ^13^C NMR (100.6 MHz, DMSO-*d_6_*) δ = 36.6 (β-CH_2_), 37.0 (β-CH_2_), 53.7 (α-CH), 54.7 (α-CH), 123.1 (CH), 125.5 (C), 125.8 (CH), 126.3 (CH), 126.4 (CH), 127.8 (CH), 128.0 (CH), 128.1 (CH), 129.0 (CH), 129.1 (CH), 129.2 (CH), 130.5 (CH), 131.7 (C), 133.3 (CH), 137.5 (C), 138.1 (C), 140.0 (C), 146.9 (C), 160.9 (C), 162.0 (C), 171.0 (C), 172.7 (C) ppm. HRMS (ESI) [M + H]^+^, *m*/*z* calculated for C_30_H_26_N_3_O_4_S: 524.1639; found: 524.1644.

##### *N*-(Benzo[*b*]Thiophene-2-Carbonyl)-l-phenylalanyl-l-phenylalanine (**10c**)

From compound **8c** (0.166 g, 0.331 mmol) in EtOH (15 mL), following the general procedure, compound **10c** was obtained (0.780 g, 50%), m.p. 231–233 °C. ^1^H NMR (400 MHz, DMSO-*d_6_*) δ = 2.93–2.99 (2 H, m, β-CH_2_), 3.06–3.11 (2 H, m, β-CH_2_), 4.44–4.49 (1 H, m, α-CH), 4.70–4.76 (1 H, m, α-CH), 7.13–7.35 (10 H, m, ArH), 7.41–7.44 (2 H, m, ArH), 7.93–7.97 (2 H, m, ArH), 8.14 (1 H, s, 3-H), 8.40 (1 H, d, *J* = 8.0 Hz, NH), 8.85 (1 H, d, *J* = 8.8 Hz, NH) ppm. ^13^C NMR (100.6 MHz, DMSO-*d_6_*) δ = 36.6 (β-CH_2_), 37.0 (β-CH_2_), 53.6 (α-CH), 54.5 (α-CH), 122.8 (CH), 124.9 (CH), 125.2 (3-CH), 125.24 (CH), 126.3 (CH), 126.4 (CH), 128.1 (CH), 128.2 (CH), 129.2 (CH), 137.4 (C), 138.2 (C), 139.1 (C), 139.4 (C), 140.2 (C), 161.3 (C), 171.2 (C), 172.7 (C) ppm. HRMS (ESI) [M + H]^+^, *m*/*z* calculated for C_27_H_25_N_2_O_4_S: 473.1530; found: 473.1533.

##### *N*-(Thieno[2,3-*b*]Pyridine-2-Carbonyl)-l-phenylalanyl-l-leucine (**11a**)

From compound **9a** (0.185 g, 0.396 mmol) in EtOH (13 mL), following the general procedure, compound **11a** was obtained (0.152 g, 87%), m.p. 168–170 °C. ^1^H NMR (400 MHz, DMSO-*d_6_*) δ = 0.85 (3 H, d, *J* = 6.4 Hz, CH_3_), 0.91 (3 H, d, *J* = 6.4 Hz, CH_3_), 1.50–1.73 (3 H, m, CH, and β- CH_2_ Leu), 2.94–3.00 (1 H, m, β-CH Phe), 3.12–3.16 (1 H, m, β-CH Phe), 4.23–4.29 (1 H, m, α-CH Leu), 4.73–4.79 (1 H, m, α-CH Phe), 7.13–7.16 (1 H, m, ArH), 7.23–7.26 (2 H, m, ArH), 7.37–7.39 (2 H, m, ArH), 7.47 (1 H, dd, *J* = 8.0, and 4.8 Hz, 5-H), 8.15 (1 H, s, 3-H), 8.38 (1 H, dd, *J* = 8.0 and 1.6 Hz, 4-H), 8.42 (1 H, d, *J* = 7.6 Hz, NH Leu), 8.62 (1 H, dd, *J* = 4.8, and 1.6 Hz, 6-H), 9.00 (1 H, d, *J* = 8.4 Hz, NH Phe) ppm. ^13^C NMR (100.6 MHz, DMSO-*d_6_*) δ = 21.4 (CH_3_), 22.9 (CH_3_), 24.3 (CHLeu), 37.1 (β-CH_2_ Phe), 39.9 (β-CH_2_ Leu), 50.5 (α-CH Leu), 54.7 (α-CH Phe), 120.5 (5-CH), 123.3 (3-CH), 126.3 (CH), 128.1 (2 × CH), 129.2 (2 × CH), 132.6 (C), 133.4 (4-CH), 138.2 (C), 139.0 (C), 148.5 (6-CH), 161.1 (C), 161.2 (C), 171.2 (C), 173.9 (C) ppm. HRMS (ESI) [M + H]^+^, *m*/*z* calculated for C_23_H_26_N_3_O_4_S: 440.1639; found: 440.1637.

##### *N*-(Thieno[2,3-*b*]Quinoline-2-Carbonyl)-l-phenylalanyl-l-leucine (**11b**)

From compound **10b** (0.233 g, 0.431 mmol) in EtOH (19 mL), following the general procedure, compound **11b** was obtained (0.120 g, 57%), m.p. 228–230 °C. ^1^H NMR (400 MHz, DMSO-*d_6_*) δ = 0.85 (3 H, d, *J* = 6.4 Hz, CH_3_), 0.90 (3 H, d, *J* = 6.4 Hz, CH_3_), 1.50–1.62 (2 H, m, β-CH_2_ Leu), 1.63–1.74 (1 H, m, CH), 2.97–3.03 (1 H, m, β-CH Phe), 3.15–3.20 (1 H, m, β-CH Phe), 4.23–4.28 (1 H, m, α-CH Leu), 4.76–4.81 (1 H, m, α-CH Phe), 7.13–7.16 (1 H, m, ArH), 7.23–7.27 (2 H, m, ArH), 7.39–7.41 (2 H, m, ArH), 7.61–7.65 (1 H, m, ArH), 7.81–7.85 (1 H, m, ArH), 8.05 (1 H, br d, *J* = 8.0 Hz, ArH), 8.15 (1 H, br d, *J* = 8.4 Hz, ArH), 8.29 (1 H, s, ArH), 8.41 (1 H, d, *J* = 8.0 Hz, NH Leu), 9.01 (1 H, s, ArH), 9.18 (1 H, d, *J* = 8.8 Hz, NH Phe) ppm. ^13^C NMR (100.6 MHz, DMSO-*d_6_*) δ = 21.5 (CH_3_), 23.0 (CH_3_), 24.4 (CH Leu), 37.2 (β-CH_2_ Phe), 40.3 (β-CH_2_ Leu), 50.9 (α-CH Leu), 54.9 (α-CH Phe), 123.2 (CH), 125.5 (C), 125.8 (CH), 126.3 (CH), 127.8 (CH), 128.1 (2 × CH), 128.9 (CH), 129.2 (2 × CH), 130.5 (CH), 131.8 (C), 133.2 (CH), 138.2 (C), 140.1 (C), 146.9 (C), 161.1 (C), 162.0 (C), 171.0 (C), 174.1 (C) ppm. HRMS (ESI) [M + H]^+^, *m*/*z* calculated for C_27_H_28_N_3_O_4_S: 490.1795; found: 490.1796.

##### *N*-(Benzo[*b*]Thiophene-2-Carbonyl)-l-phenylalanyl-l-leucine (**11c**)

From compound **10c** (0.0760 g, 0.163 mmol) in EtOH (10 mL), following the general procedure, **11c** was obtained (0.0460 g, 65%), m.p. 221–223 °C. ^1^H NMR (400 MHz, DMSO-*d_6_*) δ = 0.85 (3 H, d, *J* = 6.4 Hz, CH_3_), 0.90 (3 H, d, *J* = 6.4 Hz, CH_3_), 1.50–1.72 (3 H, m, CH and β-CH_2_ Leu), 2.94–3.00 (1 H, m, β-CH Phe), 3.12–3.16 (1 H, m, β-CH Phe), 4.23–4.28 (1 H, m, α-CH Leu), 4.71–4.77 (1 H, m, α-CH Phe), 7.12–7.16 (1 H, m, ArH), 7.22–7.26 (2 H, m, ArH), 7.37–7.44 (4 H, m, ArH), 7.92–7.99 (2 H, m, ArH), 8.17 (1 H, s, 3-H), 8.38 (1 H, d, *J* = 7.6 Hz, NH Leu), 8.89 (1 H, d, *J* = 8.8 Hz, NH Phe) ppm. ^13^C NMR (100.6 MHz, DMSO-*d_6_*) δ = 21.4 (CH_3_), 22.9 (CH_3_), 24.3 (CH Leu), 37.1 (β-CH_2_ Phe), 39.8 (β-CH_2_ Leu), 50.5 (α-CH Leu), 54.7 (α-CH Phe), 122.8 (CH), 124.9 (CH), 125.17 (3-CH), 125.2 (CH), 126.2 (CH), 126.3 (CH), 128.1 (2 × CH), 129.2 (2 × CH), 138.3 (C), 139.1 (C), 139.5 (C), 140.2 (C), 161.4 (C), 171.3 (C), 174.0 (C) ppm. HRMS (ESI) [M + H]^+^, *m*/*z* calculated for C_24_H_27_N_2_O_4_S: 439.1686; found: 439.1689.

### 3.2. Hydrogel Preparation

Conjugates **10a**–**c** and **11a**–**c** (4.00 mg of each) were weighed into sample vials and dissolved in 1 mL of water (0.4 wt%) containing NaOH (1 M, 30 μL). GdL (6.00 mg) was then added to the solution, which was left to stand overnight at room temperature. Only the conjugate **10c** formed a hydrogel, and lower concentrations were tested (Figure 4). Using 0.1 wt%, no hydrogel was formed. The CGC is 0.15 wt%.

### 3.3. Rheology

The viscoelastic characterization of hydrogels was performed at 25 °C in an MCR300 stress-controlled rotational rheometer (Anton Paar GmbH, Graz, Austria) using the Couette cell geometry (1 mL volume and 0.5 mm gap). After loading the gel-forming hydrogelator solutions into the Couette cell, a shear rate of 5 s^−1^ was applied for 1 min to the stress cell to attain sample homogenization. Gel formation kinetics were acquired over 10 h, by applying a small amplitude oscillatory shear (SAOS), with a frequency of 1 Hz and an amplitude of 0.01%, recording the shear storage (G′) and loss (G″) moduli every 100 s. Mechanical spectra were acquired for the hydrogel by performing a frequency sweep (from 100 to 0.01 Hz) while applying a constant SAOS amplitude (0.01%). Next, to test for gel break-up, a dynamic strain sweep, from 0.0001 to 100%, was performed at 1 Hz.

## 4. Conclusions

Six heterocyclic–dipeptide conjugates of Phe-Phe-OH and Phe-Leu-OH with *S* and *N*,*S*-heterocycles as *N*-capping groups were synthesized via the respective heterocyclic carboxylic acids. The heterocyclic systems, benzo[*b*]thiophene, thieno [2,3-*b*]pyridine, and thieno [2,3-*b*]quinoline cores were chosen with the aim of using them as drug carriers. The *N*-heterocycle-dipeptide conjugates were tested for gelation using the pH-lowering method, with only the *N*-benzo[*b*]thiophene Phe-Phe-OH conjugate **10c** successfully forming a hydrogel with a CGC of 0.15 wt% and pH 6.8. STEM characterization revealed nanofibers with an average thickness of 17 nm which form an entangled 3D network capable of trapping water. Rheological analysis demonstrated viscoelastic behavior (G′ = 3.03 × 10^3^ Pa; G″ = 3.28 × 10^2^ Pa) comparable to the extracellular matrix of certain human tissues, highlighting its potential for biological applications as drug delivery systems or theranostic platforms.

In contrast, the *N*-thieno[2,3-*b*]quinoline-Phe-Phe-OH conjugate **10b** formed vesicle-like nanostructures that partially merged into fibers but did not interlace enough to form a hydrogel. The *N*-capping groups containing a pyridine ring may undergo protonation, which prevents gel formation. None of the *N*-heterocycle-Phe-Leu-OH conjugates formed hydrogels.

Further investigations will focus on other *N*-heterocyclic capping groups conjugated with Phe-Phe-OH, including substituted heterocyclic rings, to evaluate their influence in the formation and properties of hydrogels for biomedical applications.

## Data Availability

The data presented in this study are openly available in the Universidade do Minho (repositoriUM) repository as part of an MSc thesis.

## Supplementary Materials

The following supporting information can be downloaded at: https://www.mdpi.com/article/10.3390/molecules30040869/s1, and contains ^1^H NMR spectra of known compounds **2a**, **2b**, **3a**, **3b,** ^1^H and ^13^C NMR spectra of the new compounds **8a**–**c**, **9a**–**c**, **10a**–**c**, **11a**–**c**, including DEPT θ = 135° spectra of compounds **9a**–**c.**
