# Supplementary material for: Synthesis of S- and N,S-Heterocycle–Dipeptide Conjugates for Supramolecular Hydrogel Formation
Source: Molecules. 2025 Feb 14;30(4):869. doi: 10.3390/molecules30040869 (PMC11858218; doi:10.3390/molecules30040869)

Supplementary Materials

Synthesis of S- and N,S-Heterocycle–Dipeptide Conjugates for Supramolecular Hydrogel Formation

Ana-Morgana G. P. Silva, Maria F. Martins, Carlos B. P. Oliveira, José A. Martins, Paula M. T. Ferreira and Maria-João R. P. Queiroz *

Supplementary Materials

1. ^{1}H NMR spectrum ($CDCl_3$, 400 MHz) of compound 2a 4

2. ^{1}H NMR spectrum ($CDCl_3$, 400 MHz) of compound 2b 5

3. ^{1}H NMR spectrum (DMSO-*d*$_6$, 400 MHz) of compound 3a 6

4. ^{1}H NMR spectrum (DMSO-*d*$_6$, 400 MHz) of compound 3b 7

5. NMR spectra of compound 8a 8

^{1}H NMR spectrum (DMSO-*d*$_6$, 400 MHz) of compound **8a** 8

^{13}C NMR spectrum (DMSO-*d*$_6$, 100.6 MHz) of compound **8a** 8

6. NMR spectra of compound 8b 9

^{1}H NMR spectrum (DMSO-*d*$_6$, 400 MHz) of compound **8b** 9

^{13}C NMR spectrum (DMSO-*d*$_6$, 100.6 MHz) of compound **8b** 9

7. NMR spectra of compound 8c 10

^{1}H NMR spectrum (DMSO-*d*$_6$, 400 MHz) of compound **8c** 10

^{13}C NMR spectrum (DMSO-*d*$_6$, 100.6 MHz) of compound **8c** 10

8. NMR spectra of compound 9a 11

^{1}H NMR spectrum (DMSO-*d*$_6$, 400 MHz) of compound **9a** 11

^{13}C NMR spectrum (DMSO-*d*$_6$, 100.6 MHz) of compound **9a** 11

DEPT θ 135 ° spectrum (DMSO-*d*$_6$, 100.6 MHz) of compound **9a** 12

9. NMR spectra of compound 9b 13

^{1}H NMR spectrum (DMSO-*d*$_6$, 400 MHz) of compound **9b** 13

^{13}C NMR spectrum (DMSO-*d*$_6$, 100.6 MHz) of compound **9b** 13

DEPT θ 135 ° spectrum (DMSO-*d*$_6$, 100.6 MHz) of compound **9b** 14

10. NMR spectra of compound 9c 15

^{1}H NMR spectrum (DMSO-*d*$_6$, 400 MHz) of compound **9c** 15

^{13}C NMR spectrum (DMSO-*d*$_6$, 100.6 MHz) of compound **9c** 15

DEPT θ 135 ° spectrum (DMSO-*d*$_6$, 100.6 MHz) of compound **9c** 16

11. NMR spectra of compound 10a 17

^{1}H NMR spectrum (DMSO-*d*$_6$, 400 MHz) of compound **10a** 17

^{13}C NMR spectrum (DMSO-*d*$_6$, 100.6 MHz) of compound **10a** 17

12. NMR spectra of compound 10b 18

^{1}H NMR spectrum (DMSO-*d*$_6$, 400 MHz) of compound **10b** 18

^{13}C NMR spectrum (DMSO-*d*$_6$, 100.6 MHz) of compound **10b** 18

13. NMR spectra of compound 10c 19

^{1}H NMR spectrum (DMSO-*d*$_6$, 400 MHz) of compound **10c** 19

^{13}C NMR spectrum (DMSO-d_6, 100.6 MHz) of compound **10c** 19

14. NMR spectra of compound 11a 20

^{1}H NMR spectrum (DMSO-d_6, 400 MHz) of compound **11a** 20

^{13}C NMR spectrum (DMSO-d_6, 100.6 MHz) of compound **11a** 20

15. NMR spectra of compound 11b 21

^{1}H NMR spectrum (DMSO-d_6, 400 MHz) of compound **11b** 21

^{13}C NMR spectrum (DMSO-d_6, 100.6 MHz) of compound **11b** 21

16. NMR spectra of compound 11c 22

^{1}H NMR spectrum (DMSO-d_6, 400 MHz) of compound **11c** 22

^{13}C NMR spectrum (DMSO-d_6, 100.6 MHz) of compound **11c** 22

1. ^{1}H NMR spectrum ($CDCl_3$, 400 MHz) of compound 2a

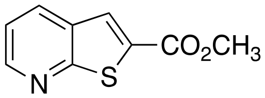

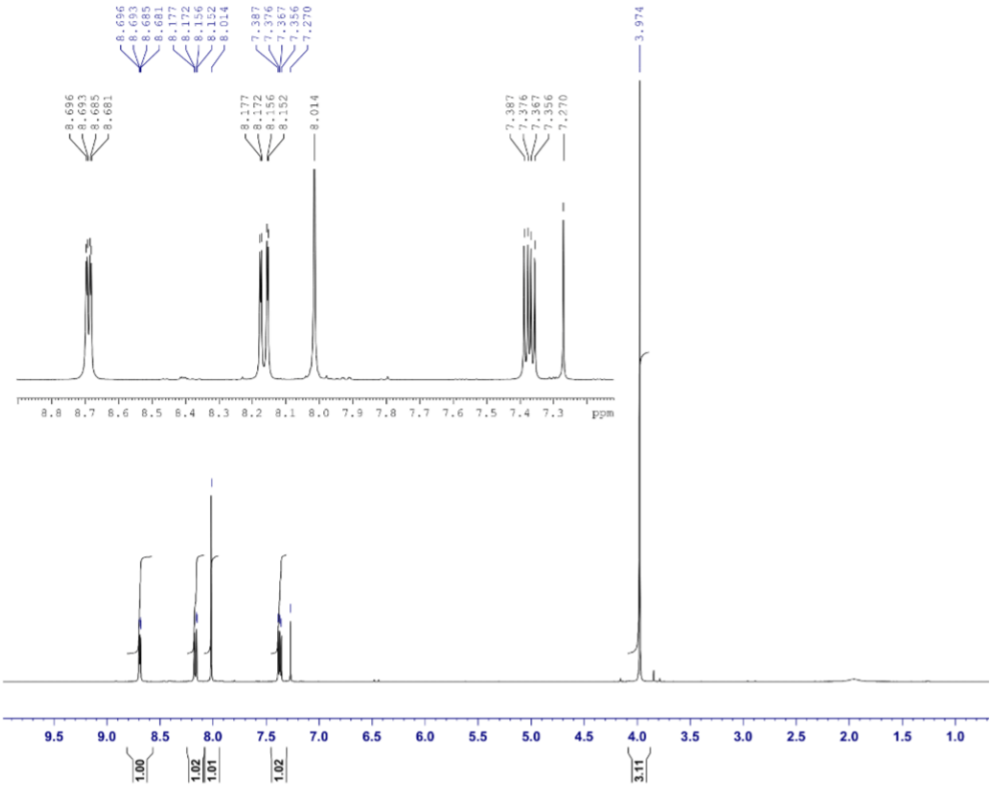

2. ^{1}H NMR spectrum ($CDCl_3$, 400 MHz) of compound 2b

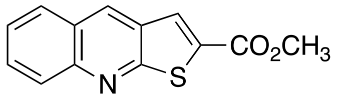

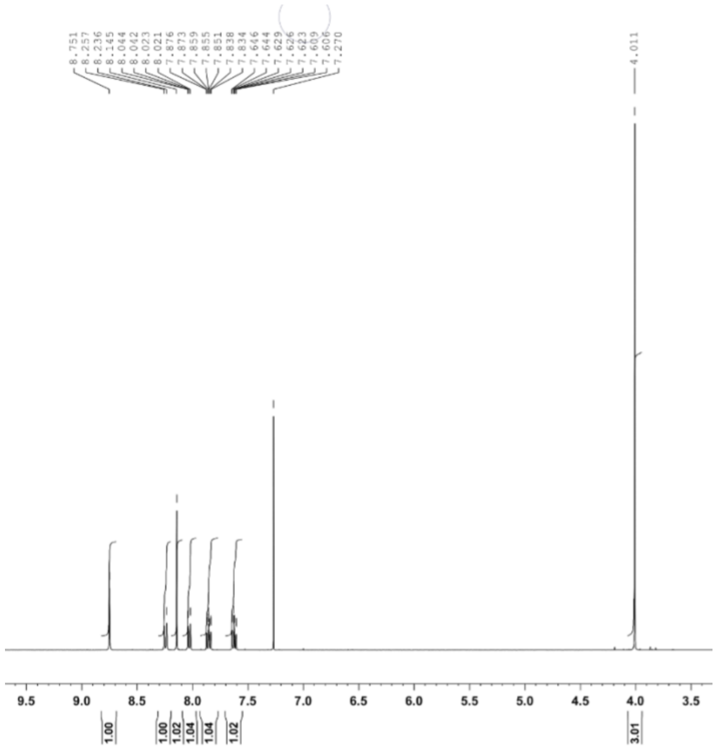

3. ¹H NMR spectrum (DMSO-d_6, 400 MHz) of compound 3a

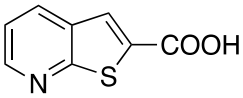

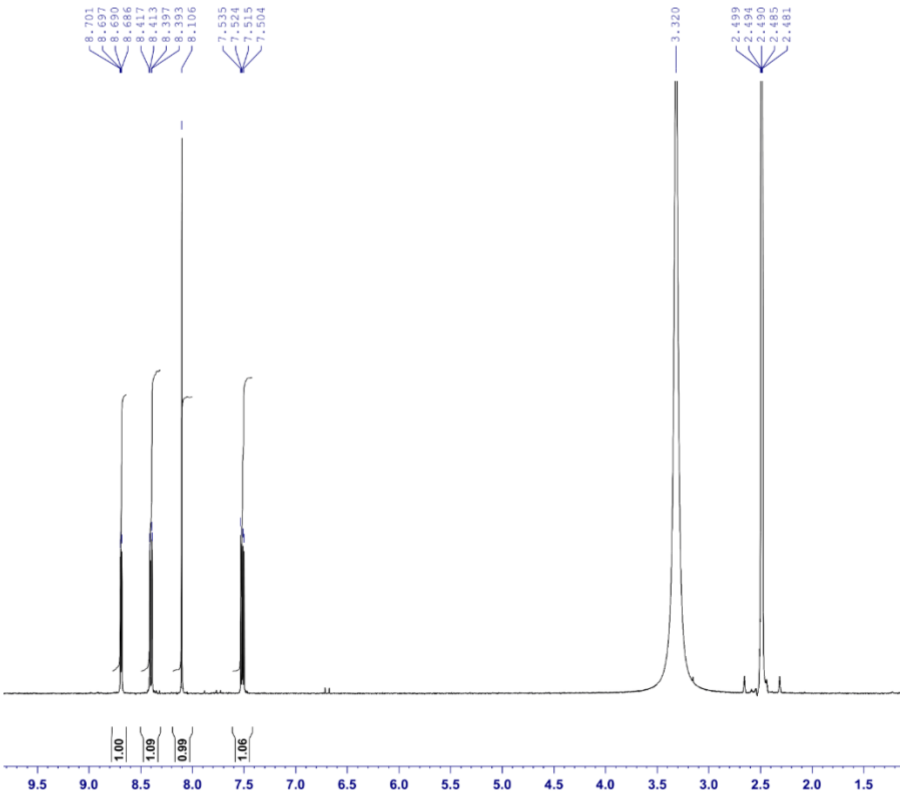

4. ^{1}H NMR spectrum (DMSO-d_6, 400 MHz) of compound 3b

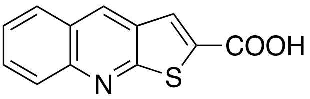

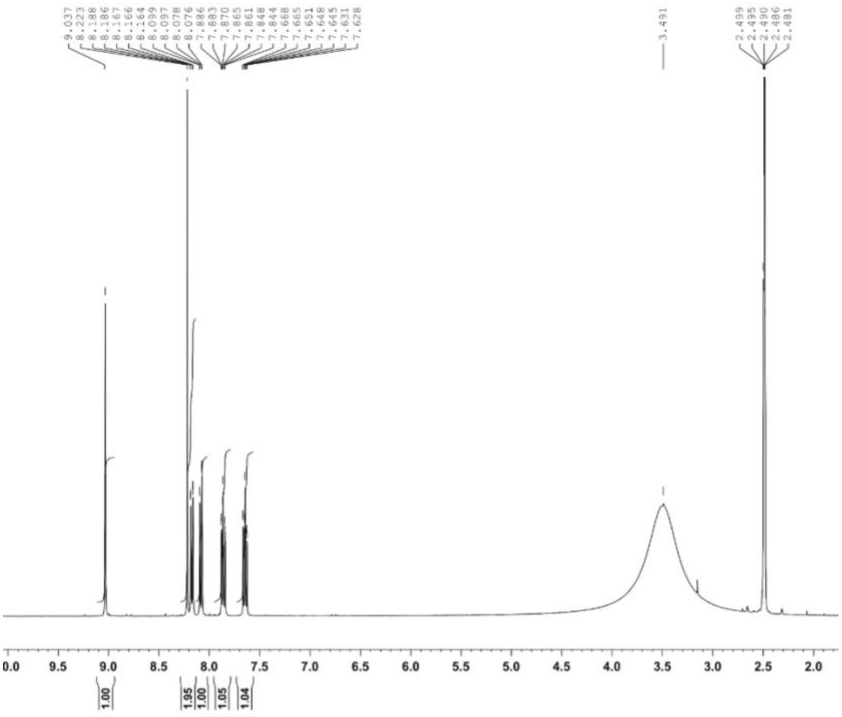

5. NMR spectra of compound 8a

^{1}H NMR spectrum (DMSO-d_6, 400 MHz) of compound **8a**

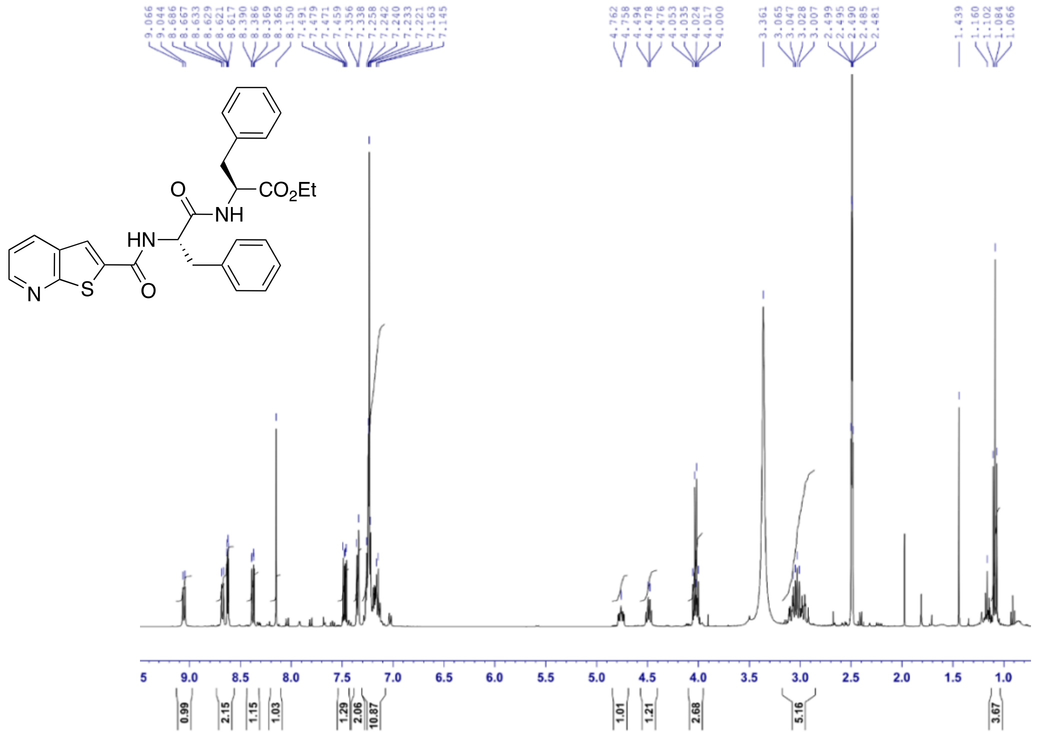

^{13}C NMR spectrum (DMSO-d_6, 100.6 MHz) of compound **8a**

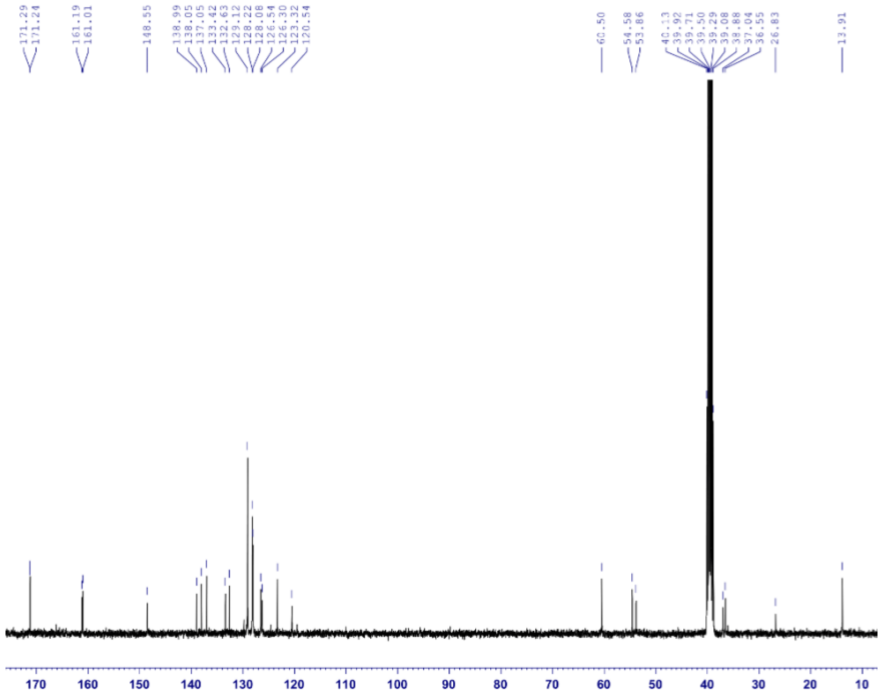

6. NMR spectra of compound 8b

^{1}H NMR spectrum (DMSO-d_6, 400 MHz) of compound **8b**

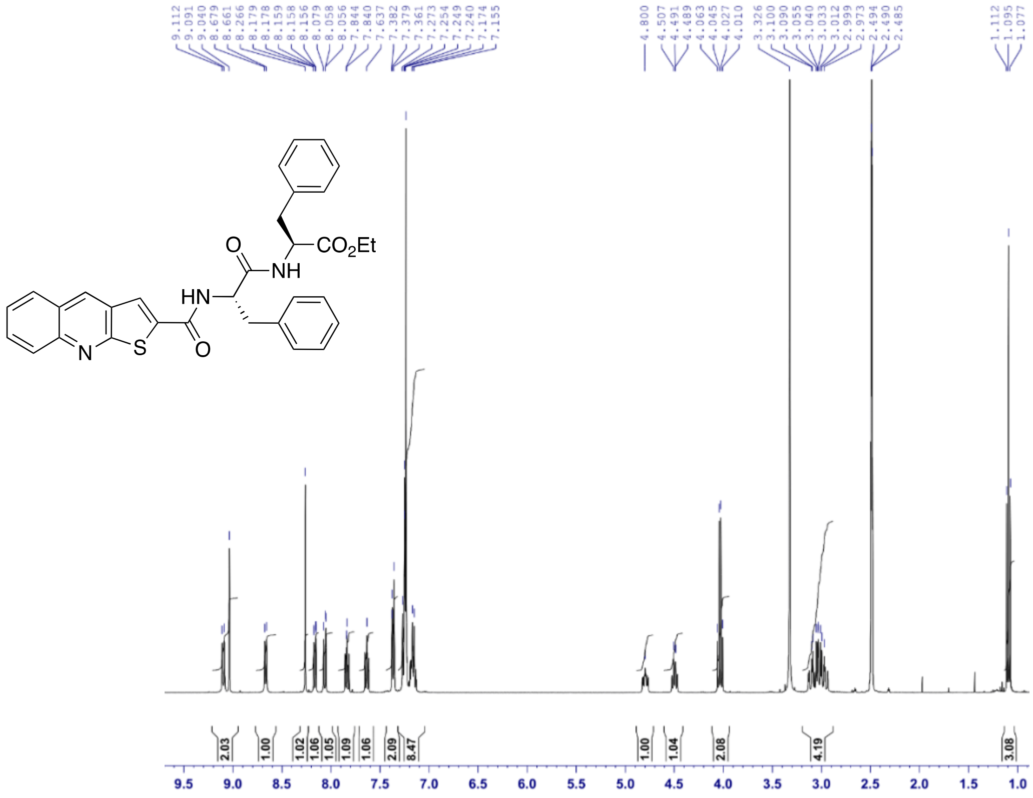

^{13}C NMR spectrum (DMSO-d_6, 100.6 MHz) of compound **8b**

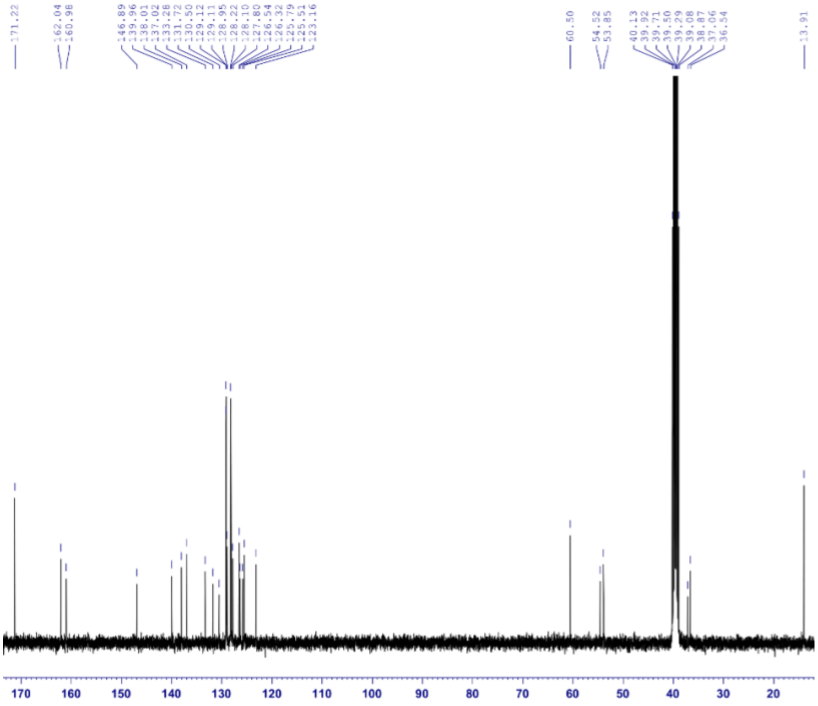

7. NMR spectra of compound 8c

^{1}H NMR spectrum (DMSO-*d*6, 400 MHz) of compound **8c**

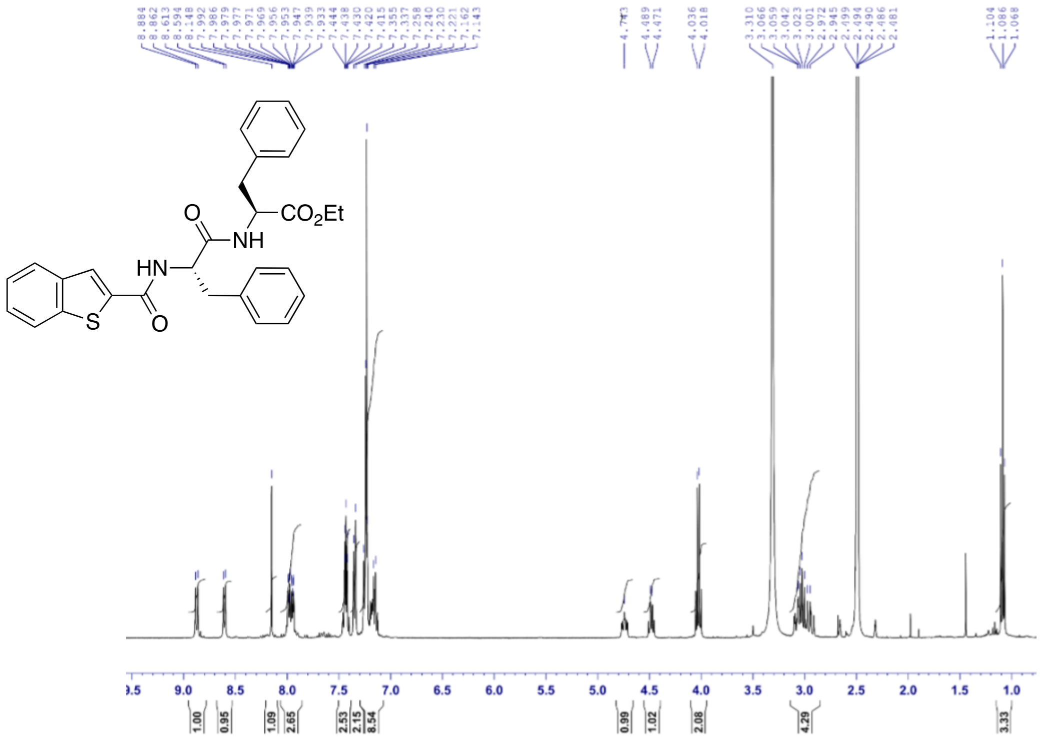

^{13}C NMR spectrum (DMSO-*d*6, 100.6 MHz) of compound **8c**

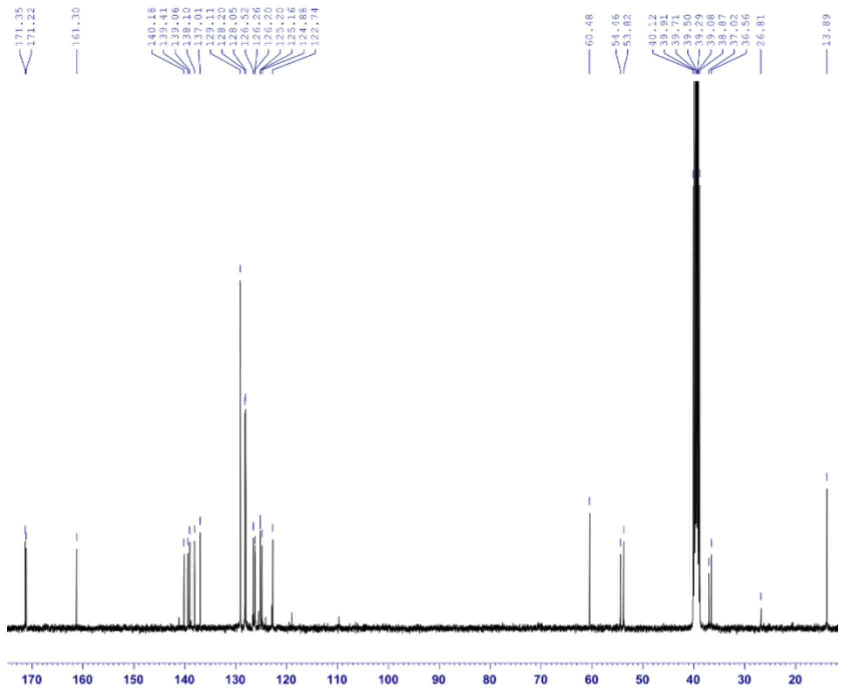

8. NMR spectra of compound 9a

^{1}H NMR spectrum (DMSO-d_6, 400 MHz) of compound **9a**

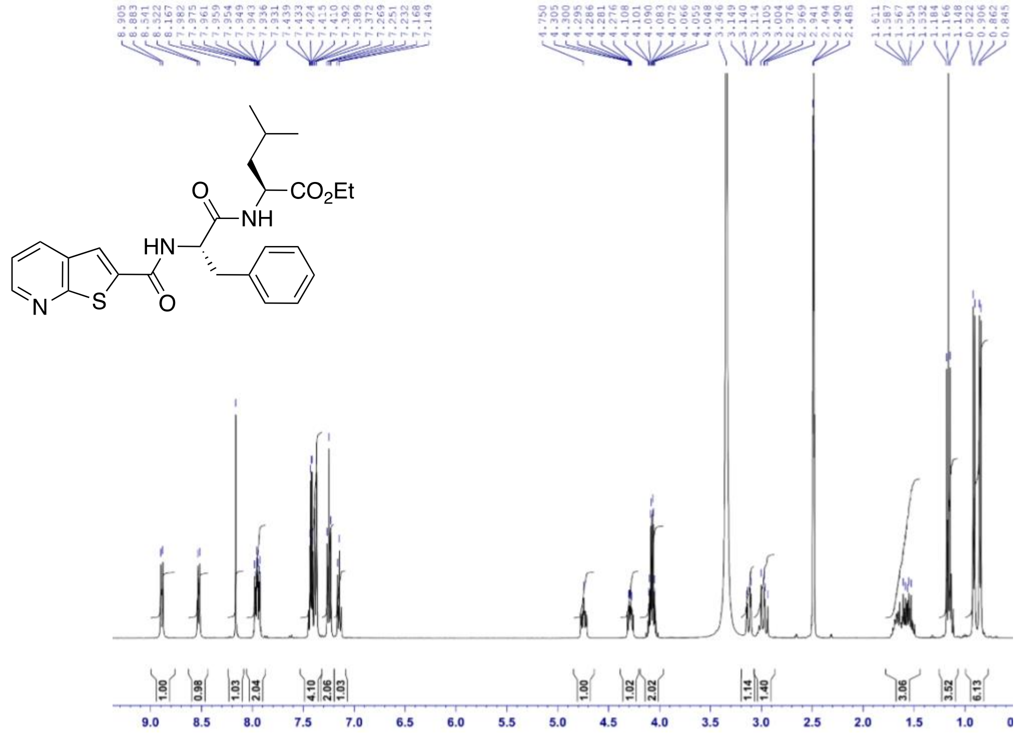

^{13}C NMR spectrum (DMSO-d_6, 100.6 MHz) of compound **9a**

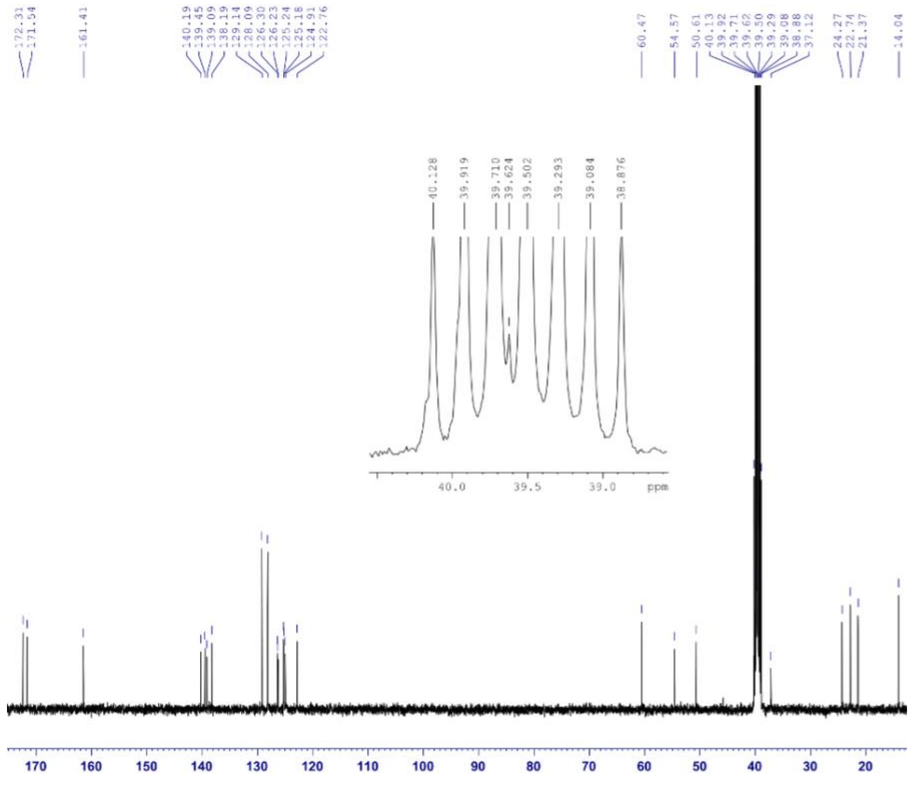

DEPT θ 135 ° spectrum (DMSO-d_6, 100.6 MHz) of compound **9a**

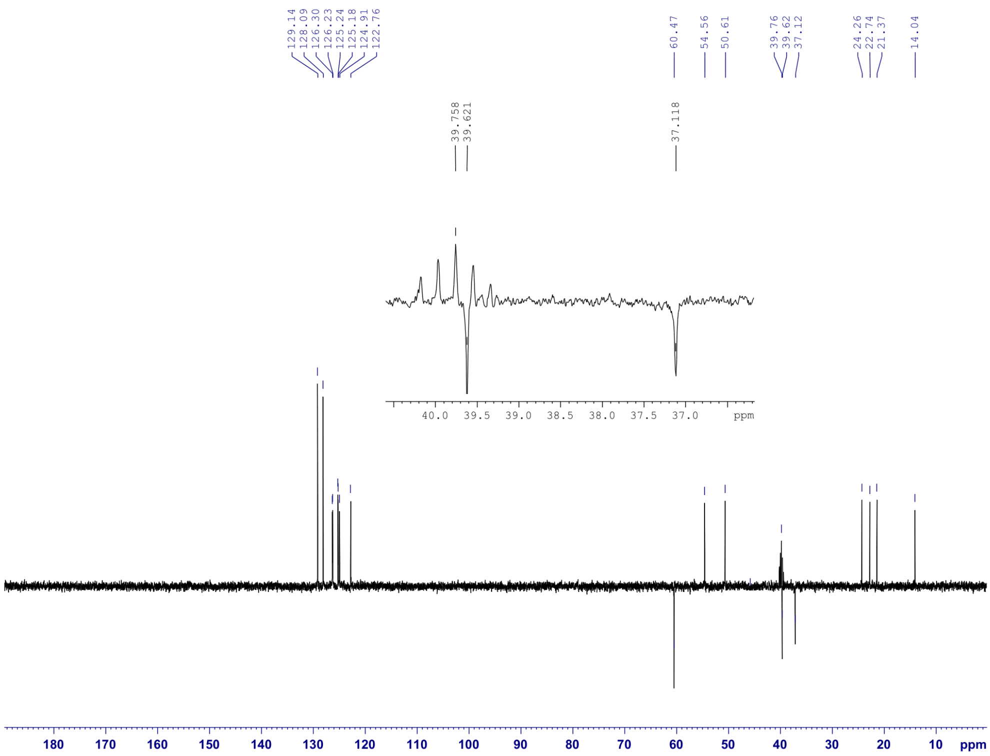

9. NMR spectra of compound 9b

^{1}H NMR spectrum (DMSO-d_6, 400 MHz) of compound **9b**

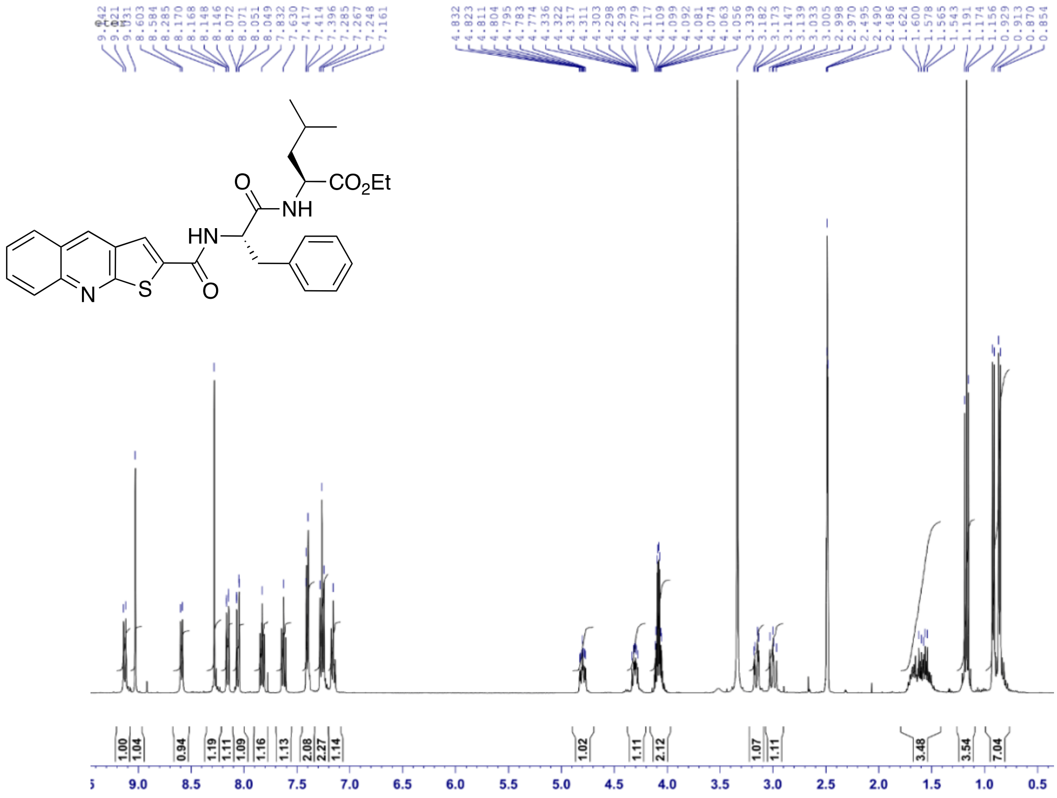

^{13}C NMR spectrum (DMSO-d_6, 100.6 MHz) of compound **9b**

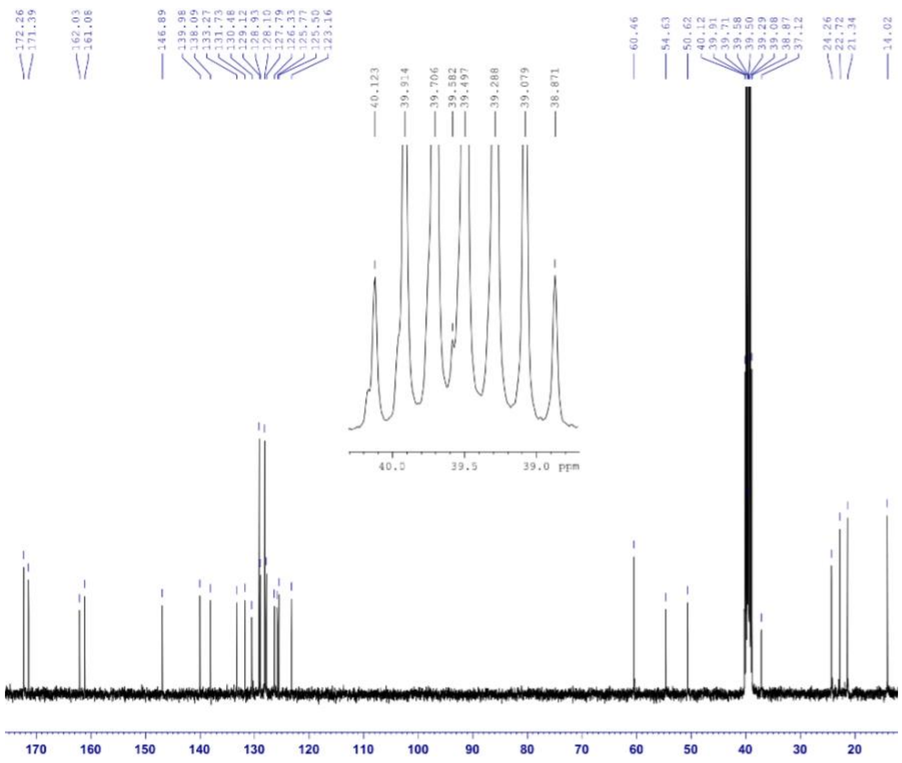

DEPT θ 135 ° spectrum (DMSO-d_6, 100.6 MHz) of compound **9b**

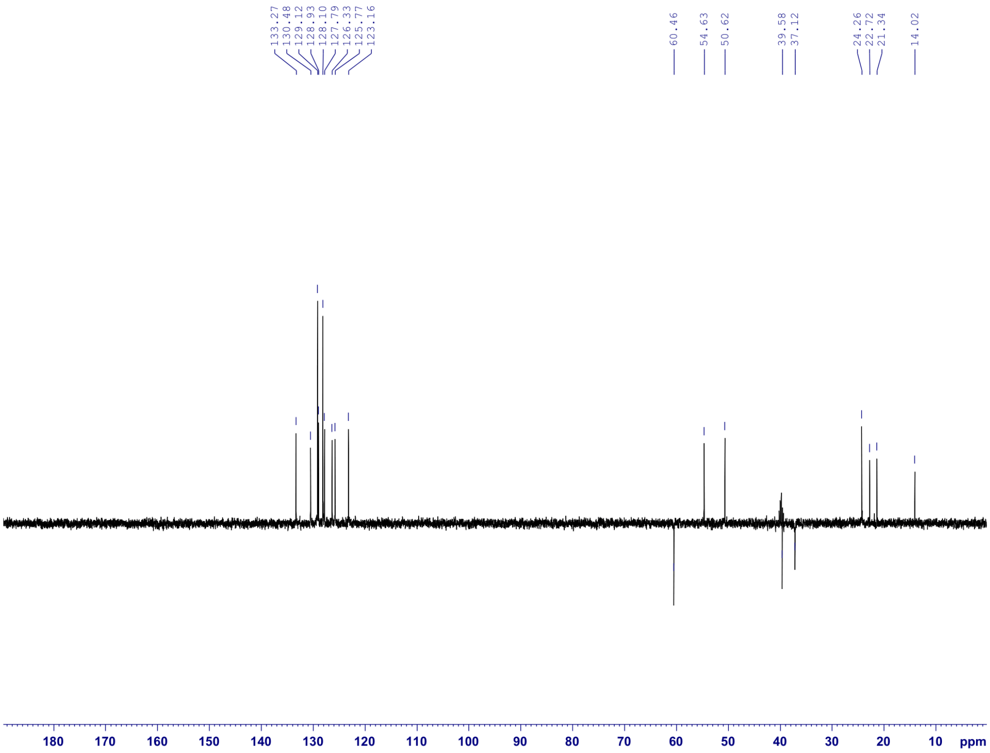

10. NMR spectra of compound 9c

^{1}H NMR spectrum (DMSO-d_6, 400 MHz) of compound **9c**

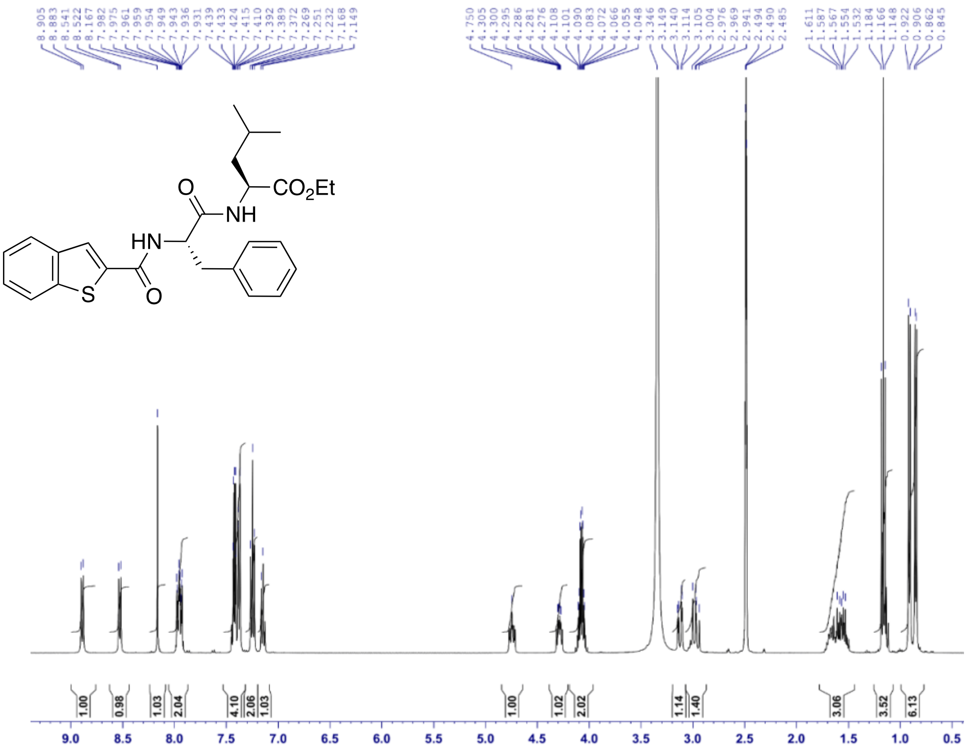

^{13}C NMR spectrum (DMSO-d_6, 100.6 MHz) of compound **9c**

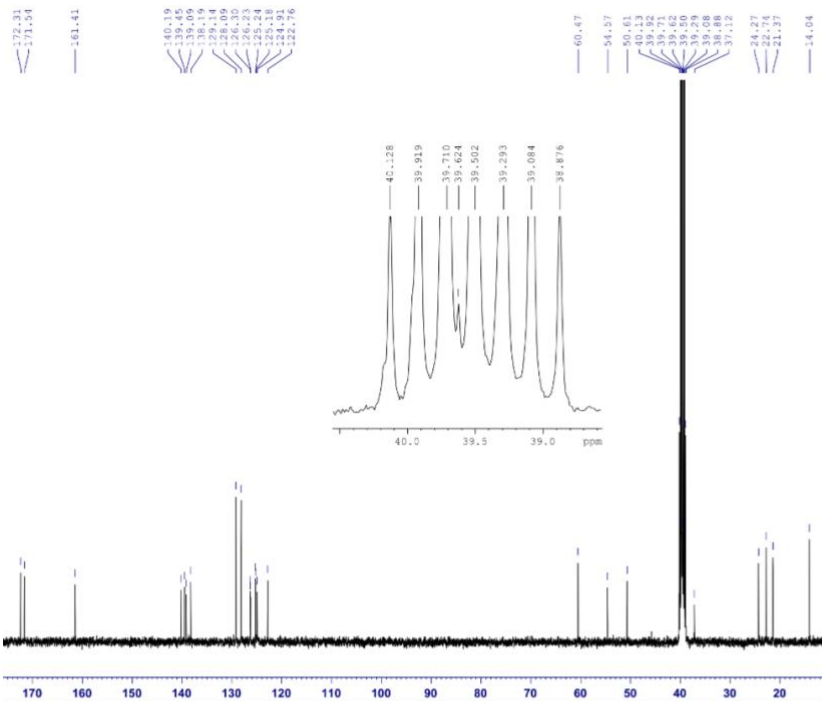

DEPT θ 135 ° spectrum (DMSO-d_6, 100.6 MHz) of compound **9c**

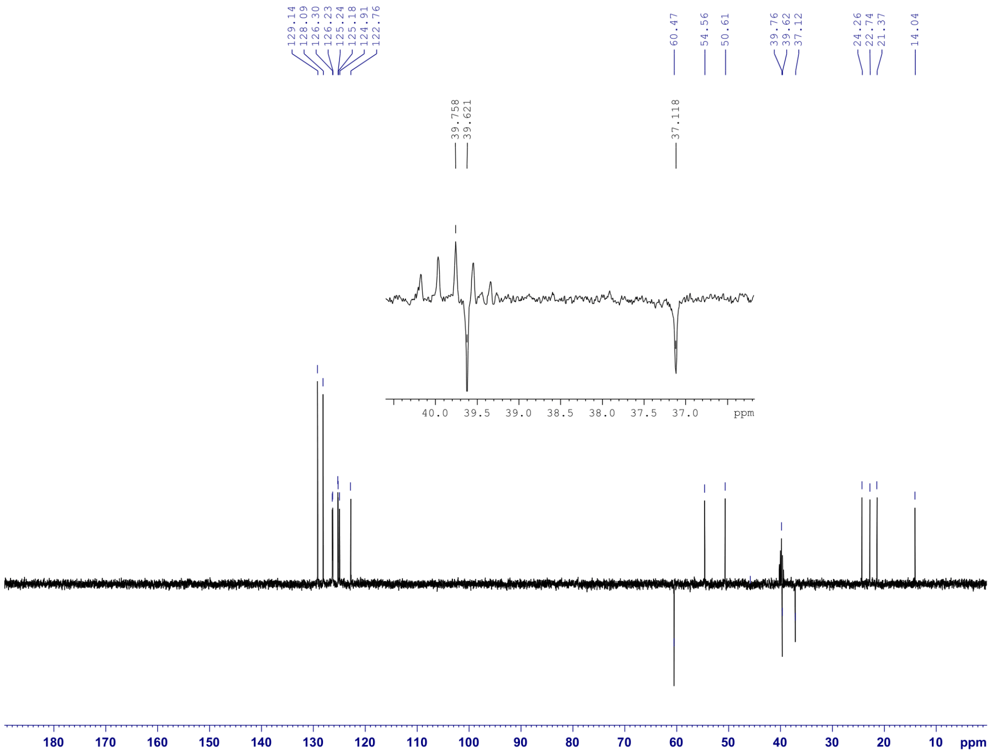

11. NMR spectra of compound 10a

^{1}H NMR spectrum (DMSO-d_6, 400 MHz) of compound **10a**

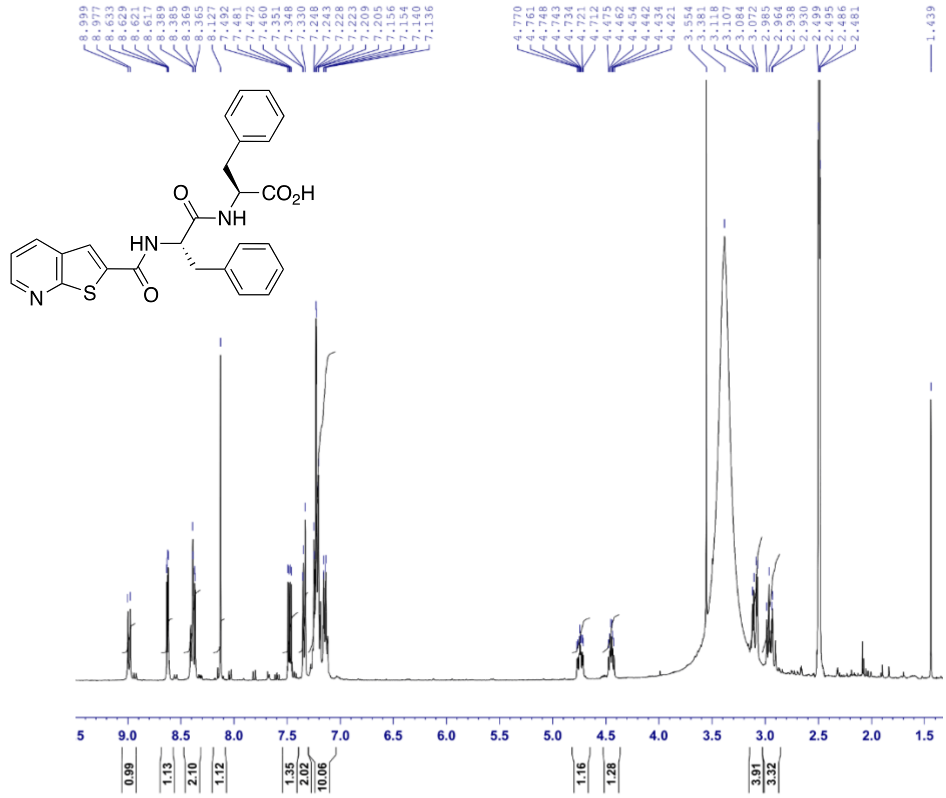

^{13}C NMR spectrum (DMSO-d_6, 100.6 MHz) of compound **10a**

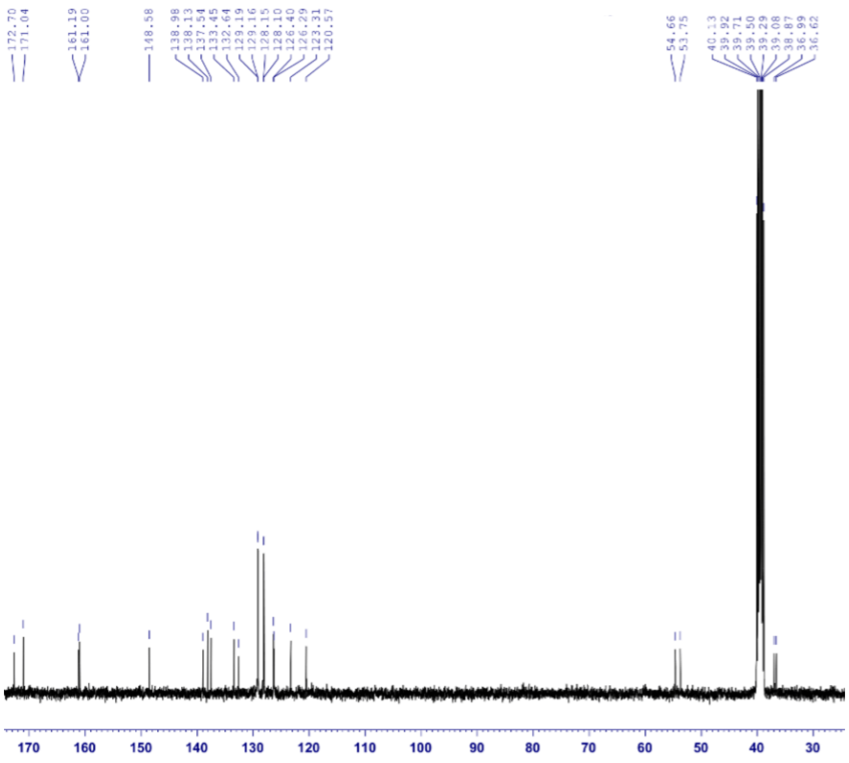

12. NMR spectra of compound 10b

^{1}H NMR spectrum (DMSO-*d*$_6$, 400 MHz) of compound **10b**

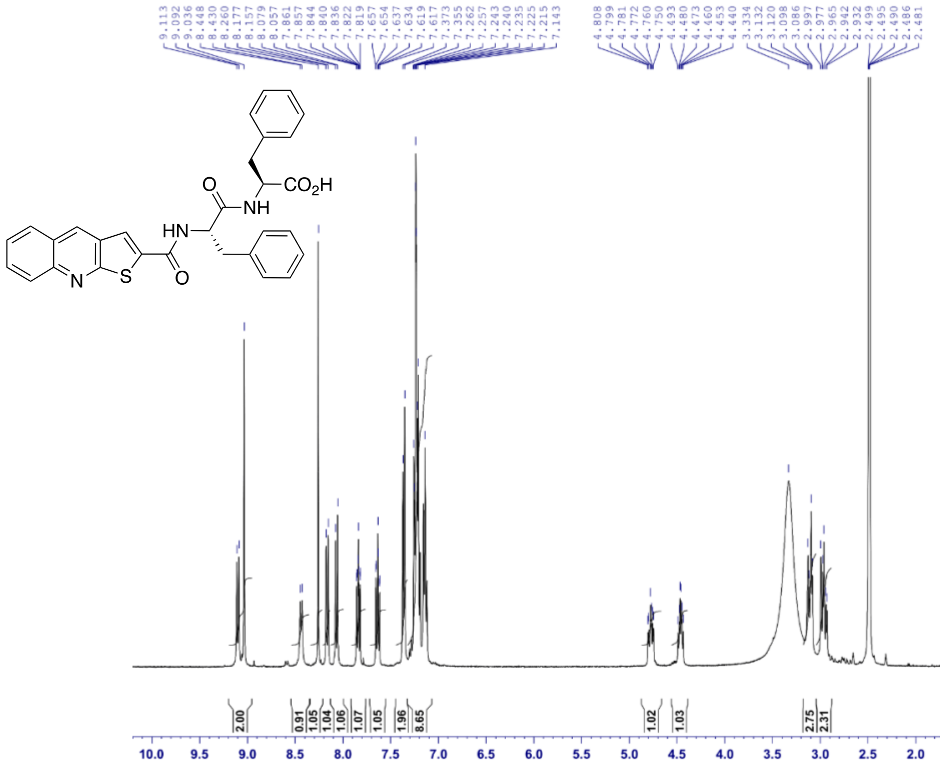

^{13}C NMR spectrum (DMSO-*d*$_6$, 100.6 MHz) of compound **10b**

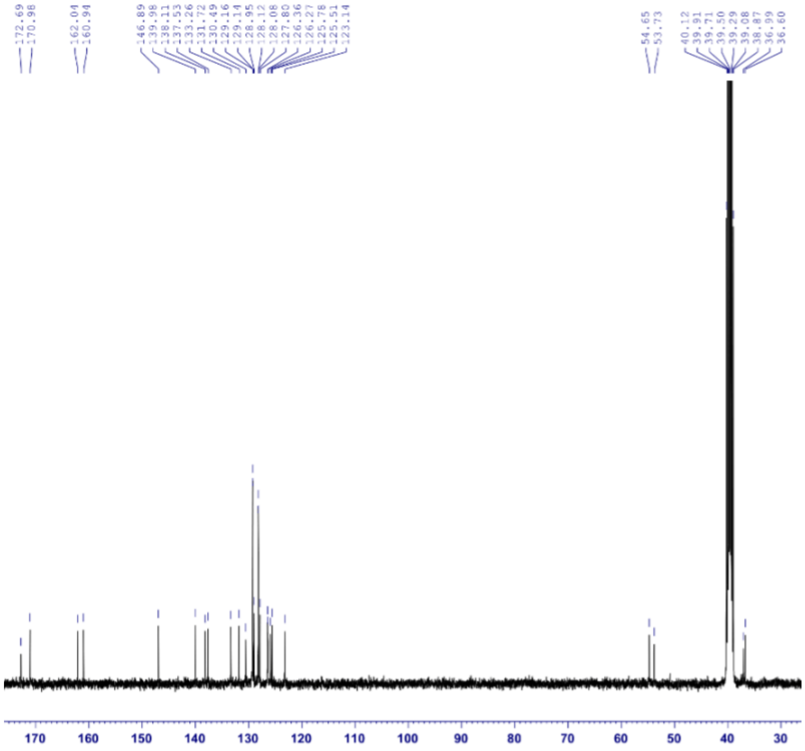

13. NMR spectra of compound 10c

^{1}H NMR spectrum (DMSO-d_6, 400 MHz) of compound **10c**

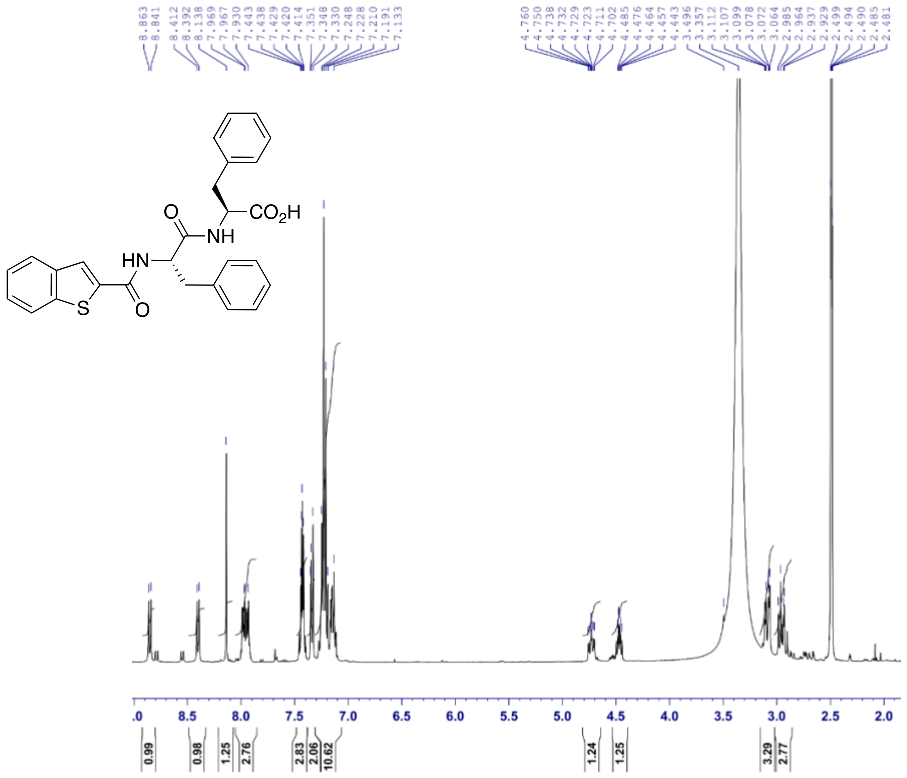

^{13}C NMR spectrum (DMSO-d_6, 100.6 MHz) of compound **10c**

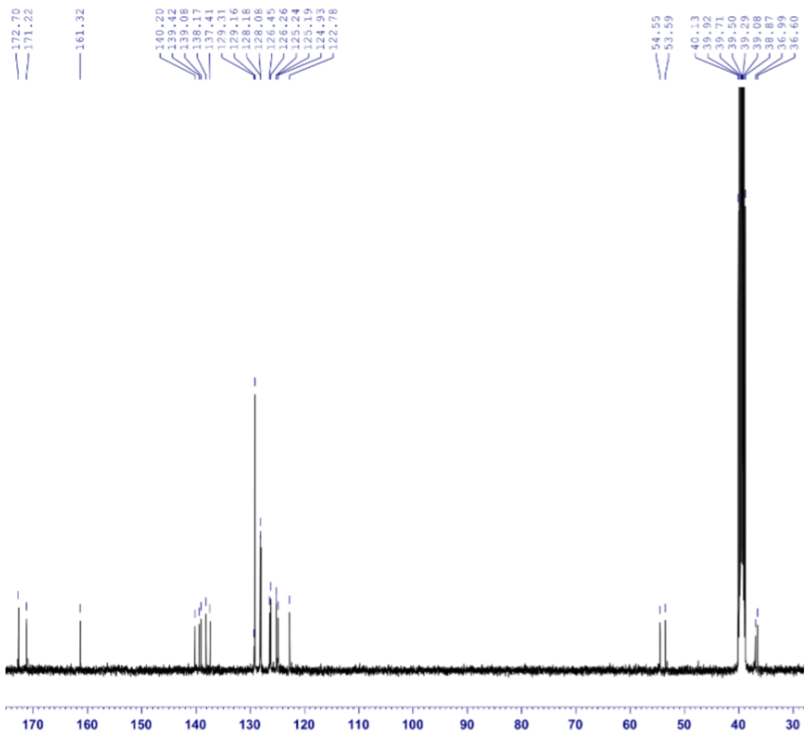

14. NMR spectra of compound 11a

^{1}H NMR spectrum (DMSO-*d*6, 400 MHz) of compound **11a**

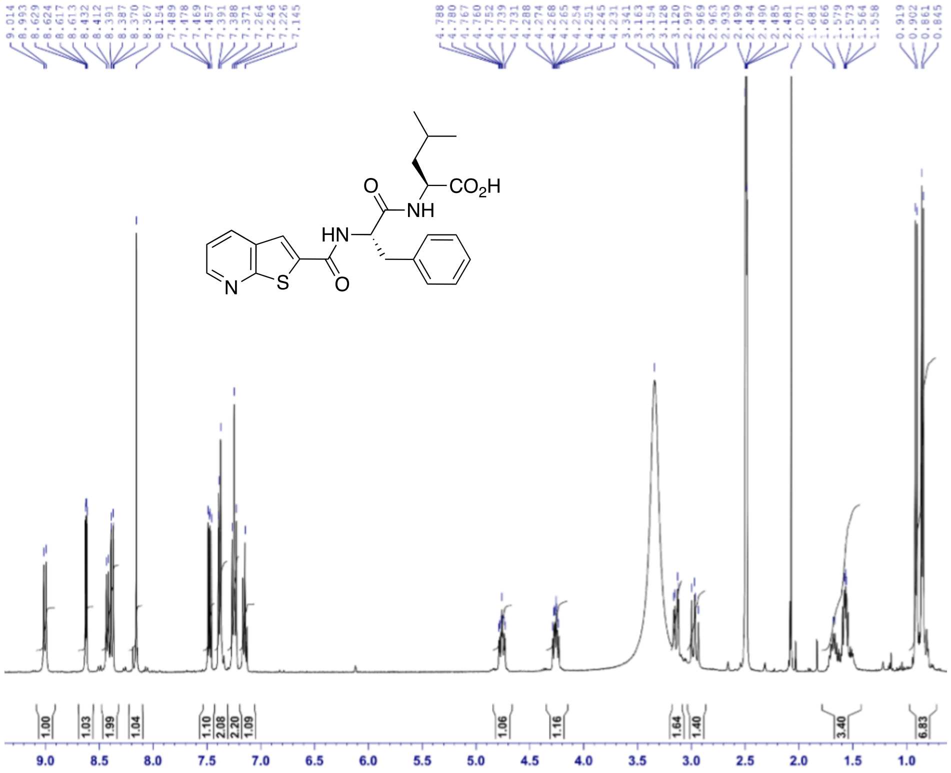

^{13}C NMR spectrum (DMSO-*d*6, 100.6 MHz) of compound **11a**

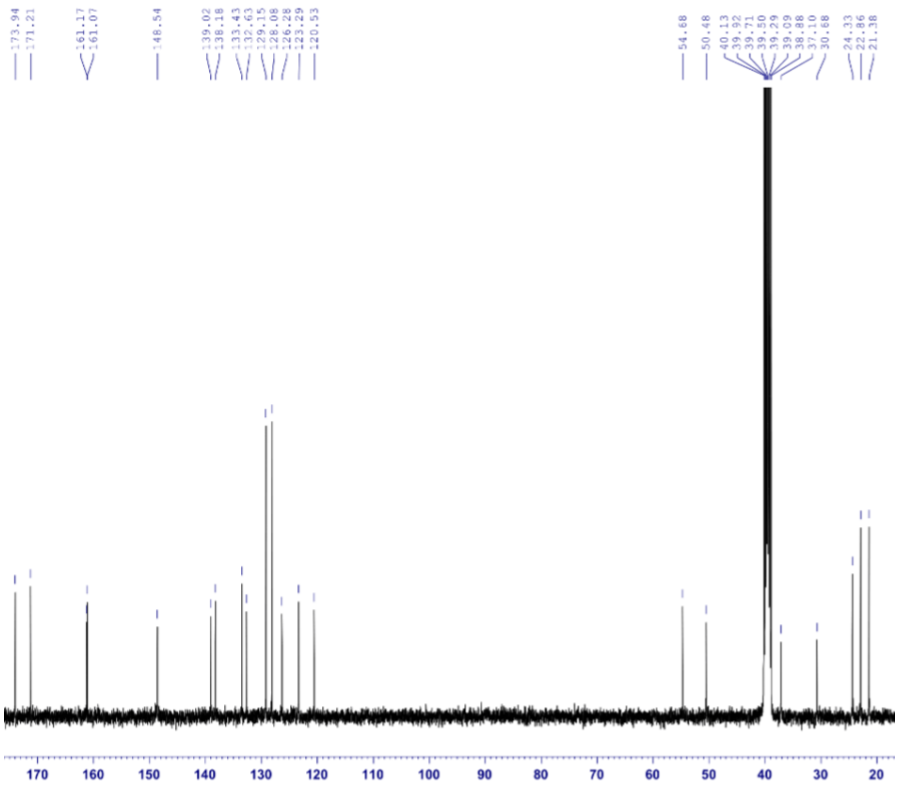

15. NMR spectra of compound 11b

^{1}H NMR spectrum (DMSO-d_6, 400 MHz) of compound **11b**

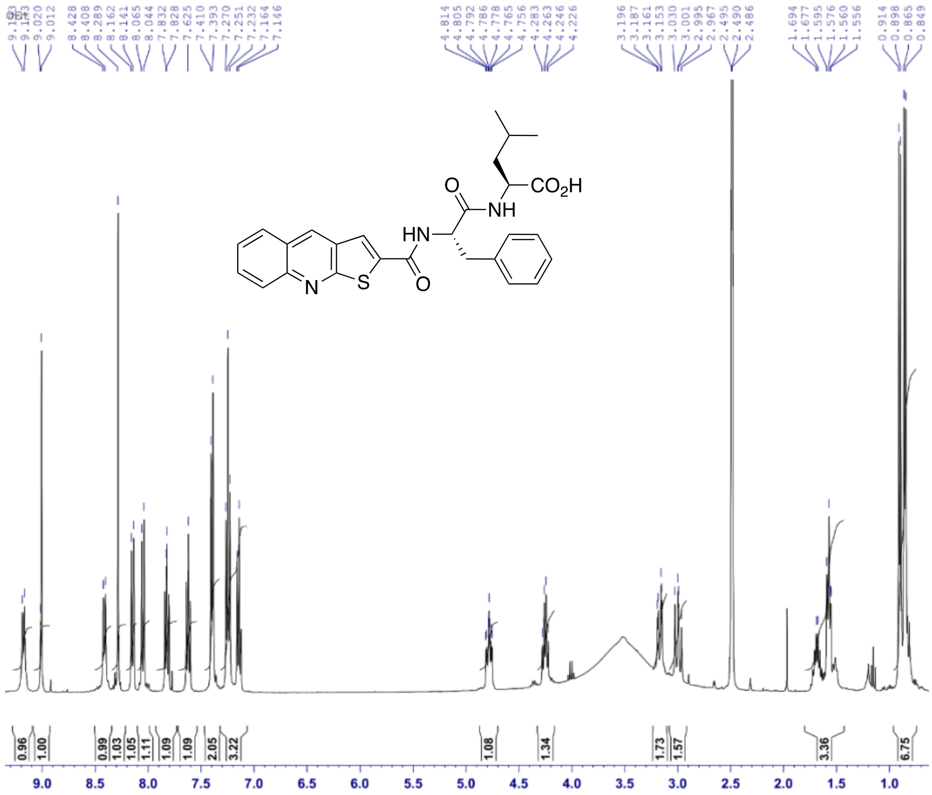

^{13}C NMR spectrum (DMSO-d_6, 100.6 MHz) of compound **11b**

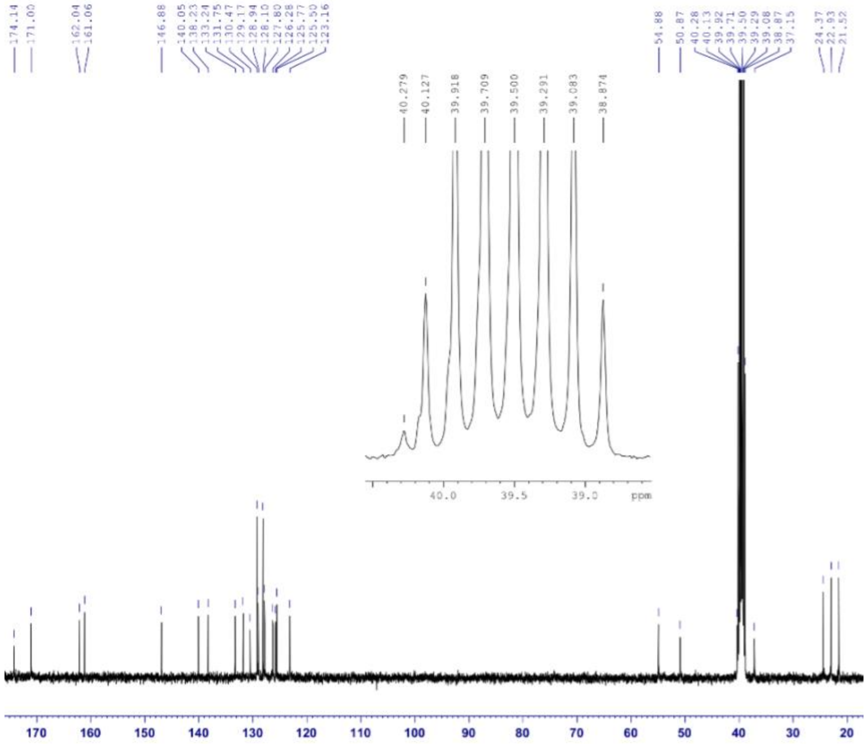

16. NMR spectra of compound 11c

^{1}H NMR spectrum (DMSO-d_6, 400 MHz) of compound **11c**

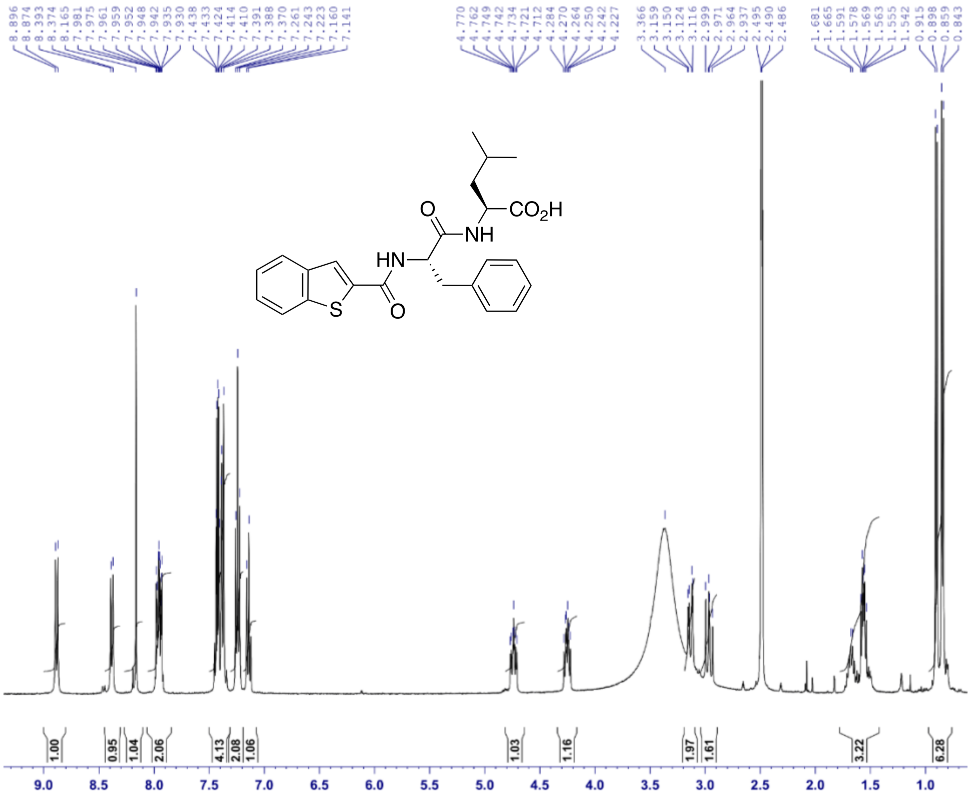

^{13}C NMR spectrum (DMSO-d_6, 100.6 MHz) of compound **11c**

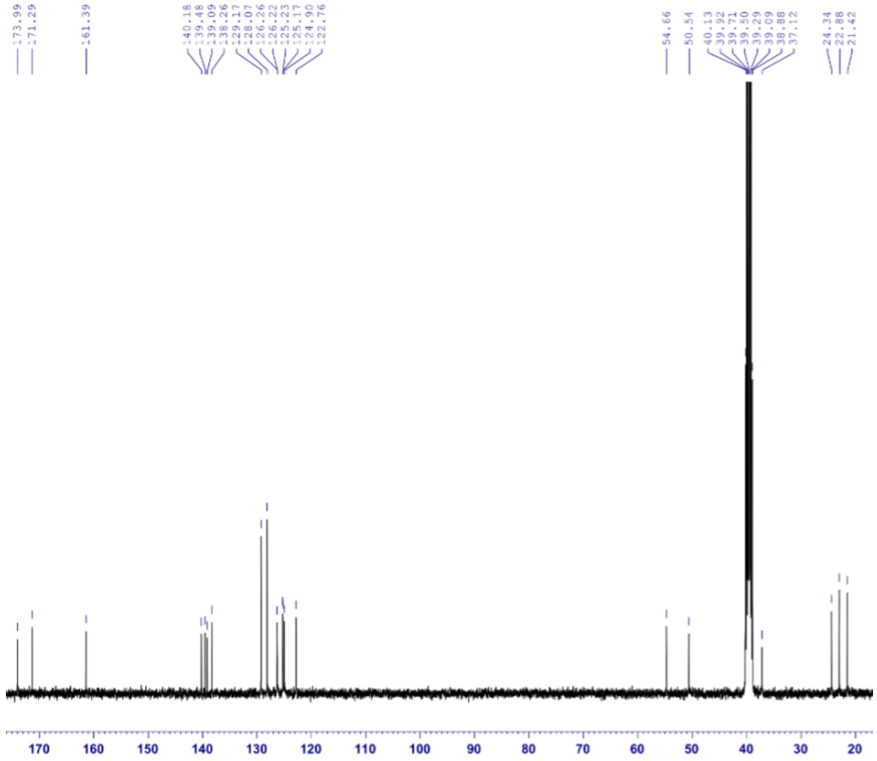

Supplement: Supplementary file 1 [file molecules-30-00869-s001.zip › molecules-3434371-supplementary.pdf]
